# Additive Manufacturing as a Design Enabler for Air-Breathing Electrochemical Devices: A PEM Fuel Cell Case Study

**DOI:** 10.3390/ma19194174

**Published:** 2026-09-29

**Authors:** Pablo A. García-Salaberri, Julia Ureña, Luis Duque, Carlos Jarava-Barrera, Antonio M. Chaparro

**Affiliations:** 1 Department of Chemical and Environmental Technology (ESCET), Universidad Rey Juan Carlos, C/Tulipán s/n, 28933 Móstoles, Spain; 2 Instituto de Investigación de Tecnologías para la Sostenibilidad, Universidad Rey Juan Carlos, C/Tulipán s/n, 28933 Móstoles, Spain; 3 Advanced Materials Department, Technology Centre of Metal-Mechanical and Transport (CETEMET), Avda. Primero de Mayo S/N, 23700 Linares, Spain; j.urena@cetemet.es; 4 Departamento de Energía, Centre for Energy, Environment and Technology Research, Avda. Complutense 40, 28040 Madrid, Spain; luis.duque@ciemat.es (L.D.); carlos.jarava@ciemat.es (C.J.-B.); antonio.mchaparro@ciemat.es (A.M.C.)

**Keywords:** additive manufacturing, air-breathing electrochemical devices, PEMFCs, geometry optimization, digital manufacturing

## Abstract

**Highlights:**

- Additive manufacturing expands the design space of electrochemical devices.
- Cathode plate geometry determines passive air-breathing device performance.
- A columnar cathode plate demonstrates manufacturing-enabled design innovation.
- The concept may extend beyond PEM fuel cells to diverse electrochemical devices.
- Digital design and additive manufacturing shape next-generation devices.

**Abstract:**

Portable air-breathing electrochemical devices, particularly polymer electrolyte membrane fuel cells (PEMFCs), offer simple, lightweight, and compact power generation by using atmospheric oxygen as the oxidant. Their performance, however, depends strongly on cathode plate geometry that governs reactant transport, thermal and water management, and mechanical integrity. Conventional manufacturing has historically restricted this architectural design space. This perspective examines how additive manufacturing (AM) enables three-dimensional geometries that can overcome these constraints. An open columnar cathode plate for air-breathing PEMFCs is used as a case study to show how manufacturing-enabled design can improve passive transport, increase the hydrogen conversion efficiency, and integrate multiple functions within a compact component. The same design philosophy can then be extended to metal–air batteries, microbial fuel cells, direct methanol fuel cells, electrolyzers, CO_2_ electrochemical reactors, and electrochemical sensors. Finally, the remaining barriers and future opportunities associated with advanced materials, computational design, topology optimization, artificial intelligence, and digital manufacturing are discussed. Overall, AM is presented as a design-enabling platform for next-generation portable electrochemical devices.

## 1. Introduction

Portable electrochemical energy conversion and storage technologies are attracting attention as society moves toward decentralized, lightweight, and energy-efficient power sources for applications ranging from consumer electronics and wearable devices to autonomous sensors, robotics, unmanned aerial vehicles, and emergency power systems. In many of these applications, system portability is determined not only by electrochemical performance but also by overall mass, volume, reliability, and manufacturing cost. Consequently, the design of electrochemical components to enhance device portability has become an increasingly important research topic, extending beyond materials development to include optimization of device architecture and system-level transport processes [1,2,3,4,5,6,7,8]. Rechargeable alkaline Zn–MnO_2_ batteries provide one example of zinc-based systems in which electrode architecture strongly affects usable capacity; porous zinc anodes have shown substantially higher discharge capacity than planar counterparts [9].

Among the strategies available to improve gravimetric and volumetric performance, passive air-breathing architectures are especially attractive because they use atmospheric oxygen directly at the cathode. Eliminating an actively supplied oxidant can reduce system mass, volume, parasitic power consumption, and balance-of-plant requirements, which is particularly valuable in small and portable devices. This approach has therefore been explored in polymer electrolyte membrane fuel cells (PEMFCs) and other electrochemical technologies in which compactness, high specific energy, and passive operation are central design requirements.

Despite these advantages, passive air-breathing electrochemical devices present distinctive engineering challenges. Hereafter, the term “air-breathing” refers specifically to devices passively supplied with ambient air, without forced convection. Because oxygen supply and product-water removal rely predominantly on diffusion, buoyancy-driven natural convection, evaporation, and gravity rather than controlled forced flow, transport phenomena become strongly coupled to cell geometry and orientation. Material selection and the design of the cathode, flow pathways, structural supports, and gas-diffusion interfaces directly influence oxygen availability, water removal, heat dissipation, electrical conductivity, and ultimately electrochemical performance. In portable systems, where auxiliary components such as compressors, fans, or active cooling systems are generally undesirable, geometric optimization becomes a key strategy for balancing these competing transport processes while preserving mechanical robustness and compactness [10,11,12,13,14,15,16,17,18,19,20,21,22,23,24,25,26,27,28,29,30,31].

However, the geometric complexity required to maximize electrochemical performance frequently exceeds the capabilities of conventional manufacturing methods. Traditional fabrication routes, including graphite machining, metal stamping, laser cutting, and injection molding, generally favor planar and relatively simple geometries that facilitate manufacturing and assembly. Although these approaches have enabled the successful commercialization of numerous electrochemical devices, they impose constraints on materials, channel design, airflow distribution, component integration, and structural optimization. As a result, many existing architectures represent compromises between manufacturing feasibility and electrochemical functionality rather than true performance-optimal designs [32,33,34,35,36,37].

Recent advances in additive manufacturing (AM) are reshaping this design landscape by allowing geometry to become an active engineering variable rather than a consequence of fabrication constraints. Layer-by-layer manufacturing enables the fabrication of complex internal channels, lattice structures, curved flow paths, integrated supports, and multifunctional components that are difficult or impossible to produce using conventional methods. Because the manufacturing process begins with a digital model, AM also integrates naturally with rapid design iteration, multiphysics simulation, topology optimization, and, increasingly, artificial intelligence (AI)-assisted design. From a materials perspective, AM provides additional opportunities through the selection and development of advanced feedstocks with tailored functional properties, including enhanced thermal conductivity, electrical conductivity, and hydrophobicity, which are particularly relevant to components such as cathode plates in electrochemical devices. Sustainability is another important dimension of AM, but its environmental benefits are process- and application-dependent. Potential advantages include the use of recycled feedstocks, recovery and reuse of unused material, part consolidation, and placement of material only where functionally required. Conversely, some AM routes—particularly energy-intensive powder-bed processes—can require substantial electricity, inert-gas consumption, support structures, powder handling, and post-processing, while not all unused feedstock can be recycled indefinitely. Accordingly, AM should not be considered intrinsically sustainable; its environmental performance should be assessed over the full life cycle and compared with conventional manufacturing for the specific material, geometry, production volume, and post-processing route [38]. When these trade-offs are favorable, AM can provide a pathway toward electrochemical architectures designed around functional and performance requirements rather than manufacturing limitations.

Against this background, this perspective uses the experimentally demonstrated open columnar cathode plate for an air-breathing PEMFC as a quantitative case study to show how AM can function as a design enabler rather than merely as a fabrication method. The case study demonstrates that manufacturing freedom can be treated as an explicit electrochemical design variable: functional and transport requirements are translated into architecture, the architecture is realized within AM constraints, and its value is assessed through measurable electrochemical and mass-specific performance. The novelty of the perspective therefore does not lie in the established ability of AM to fabricate complex geometries, but in structuring and validating this design-centered approach through a practical device in which geometric redistribution produces a measurable performance benefit. Building on this demonstrated case, the discussion formulates a broader framework linking functional requirements, transport mechanisms, architecture, performance, and manufacturability, and examines how the same reasoning can be transferred—where supported by evidence—to other electrochemical technologies. The perspective then considers scalability, economics, life-cycle implications, and specific research priorities needed to translate AM-enabled architectures from proof-of-concept demonstrations toward practical devices.

## 2. Why Portable Air-Breathing Electrochemical Devices Are Different?

Portable air-breathing electrochemical devices operate under constraints that differ fundamentally from those of conventional systems. Stationary devices can use auxiliary hardware to regulate reactant supply, temperature, water transport, and mechanical conditions; however, air-breathing portable devices must perform many of these functions passively while minimizing mass, volume, and complexity. Device architecture therefore becomes a direct determinant of both electrochemical performance and operational reliability [10,11,12,13,14,20,21,22,23,24,25,26,27,28,29,30,31,39].

The strong interactions among these requirements make geometry a critical design variable for achieving compact, lightweight, and reliable electrochemical systems without relying on auxiliary components. Importantly, these requirements are inherently interdependent and often involve competing design trade-offs. Increasing the open area available for atmospheric oxygen can enhance reactant transport but may reduce structural support; removing material can decrease mass while compromising stiffness or electrical contact; greater exposure to ambient air can facilitate heat dissipation and water removal but may also promote membrane dehydration; and increasing compression to reduce interfacial resistance can modify the porous structure of the electrode and hinder mass transport. Consequently, optimizing one function in isolation may adversely affect others. Portable air-breathing devices must therefore be conceived and optimized as fully coupled electrochemical, transport, thermal, and mechanical systems, rather than as assemblies of independently designed components.

Figure 1 brings these coupled requirements together around the central objective of effective portable air-breathing architectures. Four considerations dominate: minimizing mass and volume, enabling passive reactant and water management, providing adequate thermal regulation, and maintaining mechanical robustness. Because the same geometric features can influence several of these functions simultaneously, geometry provides a natural link among the four requirements and organizes the discussion in Section 2.1, Section 2.2, Section 2.3 and Section 2.4.

### 2.1. Mass and Volume Constraints

Portability imposes stringent limitations on both the weight and physical dimensions of electrochemical power sources. Applications such as wearable electronics, autonomous sensors, unmanned systems, and portable power supplies demand high energy output while minimizing system mass and footprint. Consequently, maximizing gravimetric and volumetric energy density is a primary objective during device design [4,5,8,40,41,42,43,44,45]. These system-level, modeling, and architectural considerations are also reflected in studies of PEMFC control, portable-fuel-cell modeling, radial and cylindrical configurations, and microfabricated fuel-cell platforms [46,47,48,49,50,51,52,53,54,55,56].

Air-breathing architectures inherently contribute to this objective by eliminating the need to store one of the reactants within the device. In fuel cells, atmospheric oxygen replaces compressed or stored oxidant supplies, reducing component count and overall system weight; however, system-level energy density still depends strongly on hydrogen storage, which can account for more than 50% of total system mass. Similarly, several metal–air battery technologies exploit oxygen from the surrounding environment to achieve theoretical energy densities substantially higher than those of conventional rechargeable batteries (approximately 300 W h kg^−1^ and 840 W h dm^−3^ for a state-of-the-art laptop Li-ion battery).

However, reductions in system mass cannot be achieved simply by removing components. The combination of lightweight materials and AM is therefore particularly attractive because it can reduce inactive mass through lightweight, functionally optimized designs while maintaining or even improving performance. Portable devices require compact integration of current collectors, bipolar or cathode plates, seals, gas-diffusion media, and structural supports while preserving efficient reactant transport and electrical conductivity. Every additional component increases assembly complexity, manufacturing cost, and parasitic mass. Therefore, multifunctional components capable of simultaneously providing structural support, reactant distribution, and electrical conduction are especially attractive for portable systems.

These considerations emphasize that maximizing practical energy density depends not only on electrochemically active materials but also on architectural design, component integration, and minimization of inactive structural mass.

### 2.2. Passive Reactant Management

One of the defining characteristics of portable air-breathing electrochemical devices is the reduction or elimination of active reactant-delivery systems. Instead of employing compressors, pumps, or blowers, oxygen delivery to the cathode relies on natural convection and molecular diffusion from the surrounding atmosphere. Although this approach significantly reduces system complexity and parasitic power consumption, passive feeding introduces new mass-transport challenges. Oxygen availability at the cathode becomes strongly dependent on the geometry of the air pathways, accessibility of the catalyst layer, and distribution of concentration gradients within the device. Poor airflow distribution may generate localized oxygen starvation, resulting in increased activation and concentration overpotentials and reduced overall efficiency [10,13,14,15,16,17,18,19,20,21,29,30,31,57,58,59,60,61,62,63,64,65,66,67].

Water management represents an equally important challenge, particularly in PEMFCs. Adequate membrane hydration is essential for maintaining high proton conductivity, whereas excessive liquid-water accumulation can obstruct gas-transport pathways and lead to cathode flooding. Conversely, excessive evaporation can promote membrane dehydration, thereby increasing ohmic resistance. These competing processes are particularly relevant in air-breathing PEMFCs, where ambient air simultaneously governs oxygen supply, heat dissipation, and water exchange with the surrounding environment [11,12,13,14,39,68,69,70,71,72,73,74,75,76,77,78,79,80,81,82,83,84,85,86]. Therefore, effective operation requires a careful balance between oxygen delivery, membrane hydration, and product-water removal without introducing additional system complexity. Experimental and modeling studies addressing hydrophilic additives, surface roughness, coatings and surface treatments, free-convection oxygen supply, water accumulation within flow channels, and neutron-radiographic visualization further highlight the strong coupling between passive airflow, electrode wetting, and water transport [87,88,89,90].

Orientation-dependent operation represents another distinctive challenge for portable air-breathing systems. Because oxygen transport, natural convection, evaporation, and liquid-water drainage may be affected by gravity, electrochemical performance can vary with device orientation. This problem is particularly relevant for wearable systems, robotics, unmanned aerial vehicles, and other mobile platforms where a fixed operating orientation cannot always be guaranteed. Architectures that reduce sensitivity to gravity by improving distributed air access and passive liquid-water evacuation are therefore highly desirable. Environmental variability, including operation at high altitude, further reinforces the need for architectures that remain robust under changing conditions [91].

### 2.3. Thermal Management

Electrochemical energy conversion inevitably generates heat, so that maintaining an appropriate operating temperature is essential for efficient and stable operation. In compact air-breathing systems, however, conventional thermal-management strategies based on forced-air or liquid cooling can undermine the advantages of portability by increasing system mass, parasitic energy consumption, and mechanical complexity [11,19,20,21,63,92,93].

Instead, heat removal must occur primarily through combinations of natural convection, thermal conduction, and radiation. The effectiveness of these mechanisms depends strongly on exposed surface area, device geometry, thermal conductivity of the structural materials, and the spatial distribution of electrochemical heat generation. Dynamic comparisons between conventional and air-breathing PEMFCs show that the lower natural-convection heat-transfer coefficients of passive configurations can lead to higher cell temperatures and slower thermal responses to changing loads.

Non-uniform temperature distributions can produce localized thermal gradients that alter electrochemical kinetics, membrane hydration, and water distribution within the cell. Because heat and water transport are closely coupled, changes in temperature may simultaneously influence evaporation, condensation, membrane hydration, and oxygen transport. Passive water and thermal management must therefore be treated as a coupled design problem rather than as independent engineering functions.

Consequently, geometrical features that increase effective heat-transfer area while maintaining reactant accessibility and water evacuation provide an important route toward improved portable electrochemical systems. Geometric optimization can also reduce structural mass. This coupling further motivates multifunctional architectures in which a structural component contributes simultaneously to mechanical support, current collection, reactant transport, thermal regulation, and mass reduction.

### 2.4. Mechanical Robustness and Flexibility

Unlike stationary electrochemical systems, portable devices may be exposed to repeated handling, changing mechanical loads, vibration, impacts, and variations in operating orientation. These conditions require sufficient mechanical robustness without substantially increasing device mass [27,94,95].

Structural components must withstand assembly compression and operational cycling while preserving sealing integrity and maintaining stable electrical contact between adjacent cell components. Mechanical deformation can alter gas-diffusion-layer thickness, porosity, contact area, and interfacial electrical resistance. Experimental measurements of PEMFC gas-diffusion layers under cyclic mechanical compression have demonstrated a strong relationship between applied compression and electrical contact resistance, together with hysteresis associated with repeated loading. Therefore, mechanical design is directly coupled with electrochemical losses rather than being merely a structural consideration.

These mechanical considerations reinforce the importance of structural design as an integral component of electrochemical engineering. Rather than serving merely as passive supports, structural elements can increasingly contribute to reactant distribution, water management, heat dissipation, current collection, compression control, and overall device reliability.

A further opportunity enabled by AM is the integration of mechanical robustness with structural flexibility, two properties that can appear contradictory but are increasingly important for portable electrochemical devices. Mechanical robustness ensures that critical dimensions, contact pressures, and structural relationships remain within the ranges required for stable, high-performance operation. Flexibility, in contrast, allows the device to accommodate external deformation while preserving its essential functional parameters. Combining these characteristics could improve the functionality, durability, and integration of portable devices, particularly in wearable, embedded, and implantable systems. AM provides a promising route through composite and multi-material architectures with locally tailored mechanical properties. Polymers, metals, and inorganic materials can be strategically combined and spatially distributed so that functionally critical regions remain mechanically robust while other regions provide controlled flexibility or conformability.

Overall, mass minimization, passive reactant management, thermal regulation, and mechanical integrity form a tightly coupled design problem. Decreasing structural mass can reduce stiffness or electrical conduction; increasing open area can improve oxygen transport while reducing contact area or strength; enhancing convective heat and water removal can alter membrane hydration; and increasing compression to reduce contact resistance can adversely affect porous transport layers. Optimizing each function independently is therefore unlikely to produce the best portable system. As a consequence, the resulting design challenge is not simply to miniaturize conventional electrochemical hardware, but to create multifunctional geometries in which structural, electrochemical, thermal, and transport requirements are addressed simultaneously. This need for architectural freedom leads directly to a second question: to what extent have available fabrication methods constrained the geometries that can be explored? Section 3 addresses that question and establishes the motivation for introducing new AM methods.

## 3. Manufacturing Constraints Have Shaped Current Designs

Electrochemical device architecture has evolved in close relationship with available manufacturing technologies. Materials science and electrochemical modeling have produced major advances in catalysts, membranes, and electrodes, yet the geometric complexity of many practical devices remains comparatively modest. In numerous cases, the architecture reflects what can be fabricated reliably and economically rather than what would be selected from transport or electrochemical considerations alone. This manufacturing influence is especially important in portable air-breathing devices because, as Section 2 showed, passive transport, thermal regulation, and structural integrity are all geometry-dependent. Established methods for bipolar plates, current collectors, flow fields, and supporting structures restrict the range of shapes that can be produced economically but also that could enhance durability and performance. Figure 2 summarizes how these restrictions favor relatively simple architectures and limit the integration of transport pathways and multiple functions within a single component.

### 3.1. Conventional Manufacturing Methods

The manufacturing of electrochemical devices relies primarily on well-established industrial processes such as graphite machining, metal forming, injection molding, laser cutting, and conventional subtractive machining. These methods have enabled reliable large-scale production while providing adequate dimensional accuracy and material compatibility [32,33,34,35,36,37].

Graphite machining remains one of the most widely used fabrication methods for bipolar plates in laboratory-scale and high-performance PEMFCs because graphite combines excellent electrical conductivity with corrosion resistance. However, machining complex three-dimensional internal features is time-consuming, generates considerable material waste, and increases manufacturing costs while introducing workshop safety concerns. As a result, flow fields are generally restricted to relatively shallow planar channels with constant cross-sections.

Metal stamping and hydroforming offer high production rates for metallic bipolar plates and are well suited for mass manufacturing. Nevertheless, these processes are constrained by forming limitations, die design, material springback, and minimum feature dimensions. Highly complex internal geometries or non-planar structures are difficult or economically impractical to fabricate using these techniques.

Injection molding has become increasingly attractive for polymer-based components owing to its low production cost and excellent repeatability at large manufacturing volumes. However, mold fabrication imposes significant geometric constraints, particularly regarding undercuts, enclosed internal channels, variable wall thicknesses, and complex three-dimensional features. Design modifications also require expensive tooling changes and may lead to the accumulation of dedicated molds, reducing flexibility and increasing development time.

Laser cutting and sheet-processing techniques are frequently employed for current collectors, flow distributors, and thin metallic components. Although these methods offer excellent precision for two-dimensional geometries, they are inherently limited in their ability to generate integrated three-dimensional architectures with multifunctional characteristics.

Collectively, these conventional manufacturing routes have established a design paradigm in which electrochemical components are fabricated individually and subsequently assembled into complete devices. Although effective and well established, this approach often increases component count, assembly complexity, sealing requirements, and interfacial losses. Moreover, traditional manufacturing methods generally rely on conventional polymeric and metallic materials whose processing routes and properties are already well established. In contrast, the development and processing of more sophisticated materials with advanced functional properties and application-specific microstructures remain comparatively underexplored. Emerging manufacturing approaches such as AM offer new opportunities to address this gap by enabling the processing of advanced materials and the tailoring of their composition, microstructure, and functional properties to meet the specific requirements of electrochemical devices.

### 3.2. Design Limitations

The constraints associated with conventional fabrication methods have had a profound influence on the geometry of electrochemical devices. Most commercial and laboratory-scale systems employ predominantly planar architectures consisting of stacked layers connected through relatively simple flow fields and mechanical fasteners. Although such configurations facilitate manufacturing and assembly, they may not provide the optimal solution for passive transport processes.

One important consequence is the limited flexibility available for designing airflow pathways. In air-breathing devices, oxygen transport depends critically on the accessibility of atmospheric air to electrochemically active regions. Conventional manufacturing generally restricts designers to straight or moderately curved channels distributed over planar surfaces, limiting opportunities to manipulate local velocity fields, diffusion distances, and reactant distribution through more sophisticated three-dimensional architectures that could substantially improve air-breathing device performance.

Component integration also remains limited. Structural supports, current collectors, flow distributors, sealing features, and mechanical reinforcements are typically manufactured as separate elements that require subsequent alignment and assembly. Every additional interface introduces potential electrical contact resistance, leakage paths, manufacturing tolerances, and assembly errors, all of which may negatively affect system efficiency and reliability.

Sealing constitutes another significant challenge. Air-breathing electrochemical devices require careful separation of reactant streams while simultaneously permitting unrestricted oxygen access from the surrounding atmosphere. Because hydrogen has exceptionally high diffusivity, anodic chambers in PEMFCs require stringent gas-tightness. Conventional layered assemblies often require multiple gaskets, compression elements, and sealing interfaces, increasing both manufacturing complexity and the risk of long-term degradation. The need to accommodate these sealing elements can itself constrain the geometry of adjacent components.

Perhaps most importantly, conventional manufacturing discourages exploration of unconventional architectures because the cost and complexity of producing new tooling can outweigh potential performance gains. Consequently, design evolution has often been incremental, focusing on modifications of established planar concepts rather than fundamentally different three-dimensional configurations that could reduce the number of separately manufactured elements while improving performance, durability, and functional integration.

### 3.3. Need for New Manufacturing Strategies and Materials

The increasing demand for lightweight, compact, and highly efficient portable electrochemical systems exposes the limitations of this traditional manufacturing paradigm. Future devices must simultaneously satisfy electrochemical, thermal, mechanical, and manufacturing requirements while minimizing component count and simplifying assembly. Achieving these objectives requires substantially advanced materials and greater geometric freedom than conventional fabrication technologies can typically provide [96,97,98,99,100].

The preceding limitations point to a change in design philosophy. Instead of selecting a fabrication route first and adapting the electrochemical component to its constraints, development can begin with functional requirements and then identify a manufacturing strategy capable of realizing the resulting geometry. Such a design-first approach is particularly relevant to portable air-breathing systems, where three-dimensional architectures can simultaneously influence oxygen transport, water removal, heat dissipation, stiffness, and mass.

Furthermore, advances in computational design and simulation—including topology optimization, generative design, multiphysics simulation, and AI-assisted optimization—are expanding the range of candidate materials and geometries that can be explored during device development. However, the practical implementation of these computationally generated architectures depends on manufacturing methods capable of reproducing highly complex geometries with sufficient precision and repeatability.

Overall, progress in portable electrochemical devices depends not only on improved materials and catalysts but also on manufacturing methods that enlarge the accessible design space. AM can complement conventional processes by enabling material–geometry combinations tailored to electrochemical function. Section 4 examines the capabilities through which AM converts manufacturing from a design constraint into a design enabler.

## 4. AM as a Design Enabler

Section 3 showed that conventional fabrication often couples geometric complexity to manufacturing difficulties. AM changes this relationship by building components layer by layer from digital models, allowing complex architectures to be produced without many of the tooling constraints associated with machining, molding, or forming. For portable air-breathing devices, this capability is valuable because it makes geometry available for direct optimization alongside electrochemical, thermal, mechanical, and transport performance.

Figure 3 organizes this expanded design space around four complementary capabilities: geometric freedom, functional integration, rapid design iteration, and digital manufacturing. Geometric freedom and functional integration broaden what can be built. Rapid iteration and digital manufacturing change how designs can be generated, evaluated, and refined. The following subsections consider these capabilities before Section 5 illustrates their combined effect through the open columnar cathode-plate case study.

### 4.1. Geometric Freedom

One of the most important contributions of AM is the geometric freedom it provides. Complex three-dimensional structures can be fabricated without many of the tooling constraints associated with machining, molding, or forming. Internal channels, overhangs, lattice structures, thin walls, curved surfaces, and spatially varying geometries can be incorporated within a single manufacturing process [97,98,99,100].

For air-breathing electrochemical devices, this flexibility enables new approaches to reactant management. Instead of relying on conventional planar flow fields, designers can develop three-dimensional airflow networks that enhance oxygen distribution while simultaneously facilitating passive water removal and heat dissipation. Local geometric features may be adapted to the expected transport conditions, allowing channel dimensions, porosity, or structural density to vary throughout the component according to electrochemical requirements.

The possibility of designing truly three-dimensional cathode architectures is particularly attractive for passive systems. Increased exposed surface area, reduced diffusion distances, and optimized airflow pathways can potentially improve reactant accessibility without increasing the overall device footprint. Such concepts are difficult to realize using conventional methods but become feasible through AM.

Moreover, AM facilitates the fabrication of bio-inspired and topology-optimized geometries that mimic naturally efficient transport networks. Branching channels, porous frameworks, controlled gradients with specific patterns, and hierarchical structures can be designed to maximize reactant delivery while minimizing pressure losses and material usage, offering entirely new opportunities for electrochemical component design.

### 4.2. Functional Integration

A second major advantage of AM is the possibility of integrating multiple functions into a single component. Conventional electrochemical systems typically consist of numerous individual parts—including flow distributors, current collectors, structural supports, spacers, and sealing elements—that must be manufactured separately and subsequently assembled. This modular approach increases assembly time, introduces manufacturing tolerances, and creates multiple electrical and mechanical interfaces [97,98,99,100].

AM enables many of these functions to be consolidated within unified architectures. Structural supports may simultaneously act as airflow distributors; conductive frameworks can provide both electrical current collection and mechanical reinforcement; sealing features may be incorporated directly into component geometries, or avoided where mechanical interfaces can be eliminated; and internal manifolds can be integrated without additional assembly operations.

Reducing component count can simplify manufacturing and improve device reliability by minimizing leakage paths, electrical contact resistance, and assembly-induced variability. For portable electrochemical systems, where compactness and robustness are critical design objectives, multifunctional components offer significant advantages over traditional layered assemblies. The combination of new materials and AM-enabled architectures may also allow structural supports to incorporate controlled flexibility, thereby improving the functionality and integration of portable devices.

Although complete multifunctional integration remains limited by the availability of printable conductive and chemically stable materials, current developments in multi-material AM suggest that increasingly integrated electrochemical devices may become feasible in the near future.

### 4.3. Rapid Design Iteration

Beyond geometric freedom, AM substantially accelerates the design process itself. Because components are fabricated directly from digital models, new prototypes can be produced without dedicated tooling or expensive manufacturing setup. This capability dramatically shortens the design-build-test cycle that characterizes electrochemical device development [97,98,99,100,101].

The on-demand fabrication of parts for rapid prototyping allows researchers to evaluate multiple architectural concepts within relatively short timeframes, facilitating systematic investigation of the influence of geometry on electrochemical performance. Parametric studies involving channel dimensions, lattice density, structural thickness, airflow pathways, or other geometric features become considerably more practical when successive prototypes can be produced simply by modifying the digital design, without requiring new molds, tooling, or extensive machining procedures. This design-to-fabrication flexibility substantially accelerates iterative development and enables a more efficient exploration of the geometric design space.

This flexibility is particularly valuable during the early stages of technology development, where design uncertainty is high and iterative refinement is essential. Rather than optimizing existing geometries incrementally, researchers can explore entirely new design concepts with relatively low manufacturing risk by developing tailored design artifacts to ensure geometry features and manufacturing tolerances before the final design.

The resulting acceleration of experimental validation also complements advances in computational modeling, allowing numerical predictions to be verified rapidly through physical prototypes and promoting increasingly integrated design methodologies.

### 4.4. Digital Manufacturing

The true potential of AM extends beyond fabrication itself. Because AM operates within a fully digital workflow, it integrates naturally with modern computational design tools, creating opportunities for data-driven optimization and automated architecture generation.

Topology optimization has emerged as one of the most promising approaches for exploiting AM capabilities. Instead of prescribing predefined geometries, topology optimization algorithms determine the optimal material distribution that satisfies multiple performance objectives while respecting manufacturing constraints. For electrochemical devices, these objectives may include maximizing reactant transport, minimizing electrical resistance, improving heat dissipation, or increasing structural stiffness simultaneously [95,97,98,99,100].

Similarly, generative design algorithms can automatically explore large design spaces by evaluating numerous candidate geometries under predefined electrochemical, transport, thermal, and mechanical constraints. When combined with multiphysics simulations, these approaches enable the systematic optimization of complex architectures that would be impractical to develop through conventional trial-and-error methods. Computational modeling can also support the prediction of material properties and processing–structure–property relationships in AM-fabricated components. Beyond optimizing geometry, simulation tools can accelerate the development and qualification of new materials and alloys by identifying suitable AM processing parameters and predicting their influence on microstructure and resulting functional properties. Such computationally assisted approaches can substantially reduce the experimental effort, material consumption, and processing time required to establish appropriate manufacturing conditions for new material systems.

AI can further accelerate this transition. Machine-learning models trained on experimental and simulation data can identify promising geometric configurations, predict electrochemical performance, and guide optimization. As these methods mature, electrochemical devices may increasingly be developed through autonomous or semi-autonomous workflows in which AI-assisted optimization, topology optimization, and AM operate as an integrated design ecosystem.

These approaches are already moving beyond purely prospective concepts. For example, Xia et al. applied topology optimization to PEMFC cathode gas channels using a transport-aware surrogate model and obtained channel architectures that outperformed conventional designs in predicted cathode transport and electrochemical performance [102]. In a complementary data-driven example, Zhou et al. generated a library of 28,348 PEMFC flow-field geometries, trained a neural network on CFD-characterized designs, and experimentally validated a machine-learning-selected flow field that increased peak power density by 10.37% relative to a conventional serpentine design [103]. These studies illustrate how topology optimization and machine learning can act directly on electrochemical-device geometry rather than serving only as future design concepts.

This convergence of digital design and advanced manufacturing fundamentally changes the role of geometry in electrochemical engineering. Rather than adapting device architectures to existing fabrication technologies, designers can optimize geometries according to electrochemical functionality and processability and then manufacture them directly using additive technologies.

These digital capabilities complete the transition from AM as a fabrication method to AM as part of an integrated design workflow. Geometry can be generated computationally, evaluated through simulation, fabricated directly, and then refined using experimental feedback.

The open columnar cathode plate for air-breathing PEMFCs provides a concrete example of this workflow: its architecture was conceived to exploit geometric freedom while addressing passive transport, structural support, and compactness simultaneously. Section 5 uses this case study to connect the general capabilities of AM with measurable electrochemical consequences.

### 4.5. Design Framework and Performance Metrics

To translate AM freedom into electrochemical value, a useful design workflow should begin with the device-level functional requirement rather than with printability alone. The proposed framework follows five linked steps: (i) define the functional requirement and operating constraint; (ii) identify the controlling physical mechanism, such as oxygen diffusion, liquid-water evacuation, ionic/electronic resistance, heat transfer, or mechanical support; (iii) parameterize the geometry that controls that mechanism; (iv) optimize the architecture while imposing AM-specific constraints such as minimum feature size, overhang, surface finish, material compatibility, and post-processing access; and (v) validate the resulting design using electrochemical, transport, structural, and manufacturing metrics. This sequence makes manufacturability part of the optimization problem rather than a filter applied after an electrochemically attractive geometry has been identified.

Performance should therefore be assessed with a metric set that spans several levels. Electrochemical metrics include current and power density, efficiency, internal or area-specific resistance, and degradation rate. Transport metrics include limiting-current behavior, effective diffusivity, pressure drop where forced flow is present, liquid-water saturation or removal rate, and thermal resistance. Architecture metrics include open-area ratio, porosity, tortuosity, characteristic transport length, surface-to-volume ratio, and inactive mass fraction. Manufacturing metrics include dimensional deviation, roughness, defect rate, part-to-part variability, build time, post-processing burden, material utilization, and cost per functional component. For portable systems, gravimetric and volumetric power or energy density provide an essential system-level check against improvements achieved only by adding structural volume or mass. Reporting these metrics together allows AM-enabled architectures to be compared on functional benefit rather than geometric complexity alone.

## 5. Case Study: Open Columnar Cathode Architecture for Air-Breathing PEMFCs

The development of a columnar cathode plate for passive air-breathing PEMFCs provides a specific example of the design principles developed in Section 2, Section 3 and Section 4. The cathode plate must compress and support the cell while allowing atmospheric oxygen to reach the gas-diffusion electrode and product water to leave the cathode, underscoring the importance of this component. These functions compete geometrically: increasing the solid fraction of the plate, i.e., the number and size of columns or the base thickness, generally improves support, clamping pressure, and dissipation of heat generated at the cathode, whereas increasing the open-space fraction facilitates air access and reduces inactive mass [10,40,75,76,77,78,79,80,81,82,83,84,85,86,94,95,104,105,106].

This balance is particularly important in air-breathing PEMFCs because forced cathode convection is absent. Oxygen supply and liquid water removal instead rely on natural mechanisms, including diffusion, capillarity, evaporation, gravity, and surface interactions, with the interplay of heat transport and rejection. Therefore, the cathode plate becomes part of the transport architecture of the cell rather than serving exclusively as a structural component.

### 5.1. From Planar Plates to Columnar Cathode Architectures

Conventional passive air-breathing PEMFCs commonly employ planar cathode plates with through-holes that expose the electrode to ambient air. Although simple, this configuration imposes a compromise between structural support and electrode exposure. Liquid water can also accumulate within individual openings, obstructing oxygen access and increasing cathode saturation [13,14,15,16,17,18,19,20,21,57,58,59,60,61,62,63,64,65,66,67,71,72,73,74,75,76,77,78,79,80,81,104,107,108,109].

The columnar architecture changes this balance by replacing a predominantly planar supporting surface with discrete columns [82]. In the experimentally investigated design, 61 columns were arranged hexagonally over a circular active area of 12.6 cm^2^, giving an aperture ratio of approximately 85%, compared with 43% for the planar perforated reference. The interconnected spaces between columns improve ambient-air access, while the column surfaces can assist liquid-water displacement through gravity, wetting interactions, and evaporation [105]. By contrast, droplets can remain trapped within the isolated openings of planar plates [110].

This geometric reorganization produced a substantial performance improvement. Under passive operation, the columnar configuration approximately doubled both the limiting current density and the peak power density relative to the planar perforated cathode, while its peak power density remained only about 15% below that of a conventional PEMFC operated with forced reactant flow. Because identical membrane electrode assemblies (MEAs) were used, the observed differences in apparent oxygen diffusivity were attributed primarily to different water-saturation levels in the cathodic porous layers [82].

These results demonstrate that reorganizing structural material around the electrode can substantially modify its transport environment without changing the electrochemically active materials. The columnar concept therefore provides the basis for further architectural optimization.

### 5.2. Evolution Toward the Open Columnar Cathode

A further development is the open columnar cathode plate illustrated in Figure 4. In this architecture, through-slots are introduced into the supporting plate, while the columns are redesigned with a slender main section and an enlarged truncated-conical distal region. The slots reduce the amount of solid material and increase communication between the cathode and ambient environment, whereas the differentiated column geometry separates the requirements for transport and mechanical contact.

The enlarged distal region provides sufficient contact area with the current collector, while the narrower column body reduces material consumption and facilitates in-plane air transport. Therefore, Figure 4 illustrates the central design principle of the open columnar cathode plate: structural material is retained where mechanical or electrical contact is required and reduced where open space can improve transport and decrease mass.

The open columnar cathode plate consequently represents more than an increase in aperture ratio. It introduces spatial differentiation of the structural material according to function: the column tips provide contact, the slender column bodies minimize obstruction, and the slots increase the open fraction of the supporting plate.

### 5.3. Performance Improvement Through Geometric Opening

The effect of this geometric evolution is summarized in Figure 5, which compares the polarization and power-density curves of three passive air-breathing PEMFC cathode configurations: a base plate without openings and with constant-section columns, modified columns with an enlarged distal region, and an open configuration combining the modified columns with through-slots. The three columnar cathode configurations compared here were manufactured from 316L stainless-steel powder using laser powder bed fusion (L-PBF).

The polarization curves show that modification of the column geometry alone produces only a modest change. The first two configurations exhibit pronounced mass-transport limitations at current densities close to 0.2 A cm^−2^. In contrast, introducing the slots extends operation to approximately 0.36 A cm^−2^ before the rapid voltage decrease associated with severe mass-transport limitation. The principal effect of opening the architecture is therefore observed in the transport-controlled region of the polarization curve.

This improvement translates directly into higher power density. As shown in Figure 5, the peak power density increases from approximately 0.107 W cm^−2^ for the reference configuration to 0.110 W cm^−2^ after modification of the columns and to 0.167 W cm^−2^ when the modified columns are combined with the slotted plate. The latter corresponds to an approximately 56% increase relative to the reference configuration. Because the main divergence between the curves occurs in the transport-controlled region, the improvement is attributed primarily to reduced cathode mass-transport limitations. The open architecture can contribute through both easier oxygen access and more effective liquid-water removal; these mechanisms are coupled because lower liquid-water saturation increases the gas-filled pathways available for oxygen transport. However, the present polarization measurements do not independently quantify the contributions of oxygen supply and water removal. Earlier experiments on related columnar cathode plates linked changes in apparent oxygen diffusivity to different water-saturation levels [82], supporting this coupled interpretation rather than a unique attribution to either mechanism.

The benefit becomes even more significant when component mass is considered. The reported mass-specific power-density metric remains approximately 0.5 mW cm^−2^ g^−1^ for the first two configurations but increases to approximately 1.5 mW cm^−2^ g^−1^ for the open columnar cathode plate (values are obtained from laboratory 2 W passive hydrogen PEMFCs of 12 cm^2^ active area and 90 g weight). Thus, Figure 5 demonstrates that opening the supporting plate provides a combined electrochemical and structural benefit: mass transport is improved while inactive material is reduced.

For portable devices, this distinction is particularly relevant. The optimum cathode cannot be identified solely from areal power density; the mass required to achieve that performance must also be considered.

### 5.4. Geometry as a Multi-Objective Design Variable

The transition from the initial columnar plate to the open architecture transforms cathode geometry into a multi-objective design problem. Column width, spacing and length, distal contact dimensions, slot geometry, and solid fraction influence different and sometimes competing requirements [37,94,95,106].

For example, larger contact areas can improve compression but increase material use and obstruction. Conversely, increasing the open fraction can facilitate transport and reduce mass but may eventually compromise mechanical integrity. The open columnar cathode plate addresses this trade-off by varying geometry according to local function rather than maintaining a uniform cross-section throughout the component. In addition, the results in Figure 5 show that geometric variables interact: modifying the columns alone provides little performance improvement, whereas combining this modification with plate opening produces a pronounced effect.

Cathode optimization should therefore consider transport, mechanical integrity, and mass simultaneously. This is especially relevant for portable systems, where improvements at the cell level must ultimately translate into higher system-level specific power.

### 5.5. AM as a Design Enabler for a Columnar Cathode

The progression from planar to columnar and ultimately open columnar architectures illustrates the role of AM as a design enabler. The relevance of AM is not simply that it can reproduce complex cathode plates, but that it allows geometric parameters to be varied with fewer of the constraints imposed by conventional machining and tooling [97,98,99,100,106].

This capability changes the design sequence. Instead of selecting a manufacturing process and adapting the cathode geometry to its limitations, functional requirements can first be translated into geometric variables. Contact area, column spacing, slot dimensions, structural mass, and open fraction can then be parameterized and iteratively optimized before fabrication.

The open columnar cathode plate illustrates this approach particularly clearly. Material is concentrated at the column tips where mechanical contact is required, reduced along the column bodies to minimize obstruction, and removed from the supporting plate where additional openings improve transport and reduce mass. Such spatial differentiation makes geometry itself an engineering variable.

Figure 4 and Figure 5 provide complementary views of the same design argument. Figure 4 shows how geometric freedom redistributes material according to local function, whereas Figure 5 shows the electrochemical and mass-specific consequences of that redistribution. Together, they demonstrate why AM is most valuable when it enables structural and transport functions to be designed concurrently rather than when it merely reproduces a conventional component.

The case study thus establishes a broader principle: manufacturing-enabled geometry can alter the transport environment of an electrochemical device without changing its active materials. Because similar trade-offs among reactant transport, product removal, structural integrity, electrical contact, and mass occur in other compact electrochemical systems, the next section examines how this design philosophy can be generalized beyond PEMFCs.

## 6. Generalization Beyond PEMFCs

Although the columnar cathode was developed for portable air-breathing PEMFCs, the underlying relationship among geometry, transport, and electrochemical performance is shared by many energy-conversion, energy-storage, and sensing technologies. The relevance of AM therefore extends beyond a single fuel-cell architecture. It provides a general means of tailoring three-dimensional structures when mass transport, thermal management, electrical pathways, and structural integration must be considered together.

Figure 6 frames this broader applicability by linking the geometry-driven design philosophy demonstrated for PEMFCs to metal–air batteries, microbial fuel cells, other fuel-cell technologies, electrolyzers, CO_2_ electrochemical reactors, and electrochemical sensors. The examples that follow are not intended as an exhaustive review and do not all represent the same level of technological maturity. In some cases, AM-enabled components or architectures have already been fabricated and experimentally evaluated; in others, the discussion represents a prospective transfer of the design principles demonstrated by the PEMFC case study. The wording below therefore distinguishes demonstrated AM applications from proposed opportunities where direct experimental evidence remains limited.

### 6.1. Metal–Air Batteries and Related Zinc-Based Systems

Among the technologies that can benefit most directly from AM are zinc–air and other metal–air batteries. Similar to air-breathing PEMFCs, these systems rely on atmospheric oxygen as one of the reactants and therefore face many of the same transport challenges associated with passive air supply [111]. More broadly, safety concerns associated with conventional Li-ion battery failure modes provide additional motivation for exploring alternative portable electrochemical energy-storage architectures [112].

The performance of metal–air batteries depends strongly on air-electrode design, where oxygen transport, catalyst accessibility, and water management occur simultaneously. Conventional planar electrode configurations impose compromises between manufacturability and transport optimization. As discussed above for PEMFCs, AM enables three-dimensional porous electrodes, hierarchical airflow pathways, and integrated current collectors that can improve reactant accessibility while maintaining light weight.

Furthermore, the ability to tailor local porosity, porosity gradients, and structural density provides opportunities to optimize electrochemical performance and mechanical stability according to the requirements of different zinc-based battery chemistries. The importance of electrode architecture is evident in rechargeable alkaline Zn–MnO_2_ systems, where porous zinc anodes reached approximately 220 mAh g^−1^ compared with 130 mAh g^−1^ for planar zinc anodes [9], illustrating how three-dimensional electrode design can alter usable capacity even outside AM-specific demonstrations. A direct AM-specific example is provided by Zhu et al. [113], who used direct ink writing to fabricate freestanding, template-free three-dimensional metallic Zn anodes with programmable lattice architectures. The printed structures combined approximately 200 μm-scale features with controllable porosity and directional transport pathways. In Zn||NiOOH cells, a periodic porous Zn lattice operated for more than 650 cycles at 25 mA cm^−2^, delivering an average areal capacity of 11.89 mAh cm^−2^; increasing the printed electrode thickness enabled an areal capacity of 85.45 mAh cm^−2^. Figure 7 illustrates the direct-ink-writing route and benchmarks the areal capacity of the printed Zn architecture against previously reported Zn electrodes. Although this system is an alkaline Zn battery rather than a Zn–air battery, it provides direct experimental evidence that AM can control Zn-electrode architecture, active-material loading, and transport pathways. On the air-electrode side, Men et al. [114] demonstrated the complementary application of AM specifically to zinc–air batteries by fabricating xylem-inspired multichannel cathodes. The aligned channels were designed to sustain oxygen transport and enlarge the three-phase reaction region, and the optimized BMC-600 cathode achieved a peak power density of 170.1 mW cm^−2^ and an open-circuit voltage of 1.51 V. Together, these studies show how AM can act on both zinc-electrode architecture and air-cathode transport structures, while the relevant performance metrics and electrochemical mechanisms remain system-specific.

### 6.2. Microbial Fuel Cells

Microbial fuel cells constitute another promising application in which geometry significantly influences electrochemical behavior. In these systems, electrode architecture affects microbial colonization, nutrient transport, metabolite removal, and electron transfer between microorganisms and conductive surfaces [115,116,117].

Traditional electrode-manufacturing techniques generally limit the complexity of internal pore structures and the accessible surface area. AM has been used to fabricate electrodes with controlled three-dimensional geometries, interconnected porous networks, and customized architectures designed to enhance biofilm development while facilitating mass transport. A quantitative example is the extrudable activated-carbon/alginate cathode reported by Theodosiou et al., which produced a maximum power of 286 μW, compared with 98 μW and 85 μW for two conventional comparison cathodes [117]. This result illustrates that AM-enabled material–geometry integration can directly affect device performance, although the relevant metric and reference architecture remain technology-specific. Figure 8 summarizes these values and provides a quantitative benchmark beyond the PEMFC case study.

The digital nature of AM could also enable electrode geometries to be adapted to different microbial communities, reactor configurations, or wastewater treatment applications, expanding the design space available for bio-electrochemical systems.

### 6.3. Direct Methanol Fuel Cells and Other Fuel Cell Technologies

Direct methanol fuel cells (DMFCs) differ from air-breathing PEMFCs in fuel delivery and operating characteristics, but they share the need for efficient transport management and compact system integration. Bipolar plates, flow distributors, and reactant manifolds remain central design elements whose geometry strongly influences device performance. Recent studies further emphasize the sensitivity of liquid-feed and related fuel-cell architectures to transport-layer configuration, assembly compression, and geometry-dependent mass transport [118].

AM could enable these components to be redesigned using integrated, geometrically optimized manifolds, advanced flow fields, and multifunctional structural elements that reduce component count while improving reactant distribution. Similar principles can be extended to other low-temperature fuel-cell technologies in which geometric optimization contributes directly to electrochemical efficiency. The same architecture-centered perspective applies to polymer-electrolyte water electrolyzers, where the porous-transport-layer/catalyst-layer interface strongly influences transport and electrochemical performance [119,120]. For example, L-PBF titanium porous transport layers with controlled anisotropic pores and through-plane microchannels have demonstrated a 21% reduction in ohmic resistance relative to conventionally manufactured counterparts, directly linking printable pore architecture to interfacial electrical performance [119].

### 6.4. Electrochemical Sensors

Electrochemical sensing platforms can also benefit from geometric customization, particularly where miniaturization, portability, and application-specific integration are required. AM has enabled the fabrication of customized sensor housings, microfluidic channels, electrode supports, and integrated analytical platforms tailored to individual sensing applications [97,98,99,100,120].

Because digital manufacturing allows rapid adaptation of device geometry, sensor architectures can be optimized for sample handling, fluid distribution, electrode placement, or integration with wearable and portable electronic systems. These capabilities are particularly attractive for point-of-care diagnostics, environmental monitoring, and distributed sensing technologies.

### 6.5. A Common Design Philosophy

Across these examples, a common design principle emerges: electrochemical performance depends not only on active materials and reactions but also on the architecture through which reactants, products, heat, and electrical current are transported. AM is therefore most consequential when it is used to tailor those pathways and consolidate functions rather than simply replacing an existing fabrication step.

From this perspective, AM should not be viewed simply as a flexible fabrication method but as an enabling technology that expands the electrochemical design space. By removing many of the geometric constraints imposed by conventional manufacturing, AM allows researchers to explore architectures specifically tailored to transport phenomena, multifunctional integration, and system-level optimization. These quantitative examples also suggest how cross-technology benchmarking should be interpreted. The open columnar PEMFC cathode plate increases peak power density by approximately 56%; the direct-ink-written Zn lattice anode operates for more than 650 cycles at 25 mA cm^−2^ with an average areal capacity of 11.89 mAh cm^−2^, while a thicker printed architecture reaches 85.45 mAh cm^−2^; the 3D-printed BMC-600 zinc–air cathode reaches 170.1 mW cm^−2^ while explicitly using multichannel geometry to promote oxygen transport; the AM-compatible microbial fuel-cell cathode raises maximum power from 85–98 μW for the comparison cathodes to 286 μW; and the L-PBF electrolyzer porous transport layer reduces ohmic resistance by 21%. Because these values represent different physical mechanisms, units, operating conditions, and normalization bases, they should not be collapsed into a single universal AM improvement factor. Instead, this section demonstrates the value of reporting architecture-specific metrics together with a clearly defined reference design.

The examples in Figure 6, Figure 7 and Figure 8 show that the columnar-cathode design philosophy can be generalized, but they also expose a practical question: can AM-enabled concepts meet the electrical, durability, manufacturing, and cost requirements of real devices? Section 7 turns from design opportunity to implementation, identifying the barriers that must be overcome before these architectures can move from laboratory demonstrations to broader deployment.

## 7. Remaining Challenges

Despite the significant opportunities offered by AM, its widespread implementation in portable air-breathing electrochemical devices remains limited by challenges at both the device-performance and manufacturing levels. As summarized in Figure 9, these barriers include achieving sufficient power density and low internal resistance, maintaining long-term stability, overcoming material and process limitations, scaling production at acceptable cost, establishing standardized manufacturing and testing procedures, and ensuring reliable operation under real-world environmental conditions. These issues are interconnected: material selection and printing quality influence electrical losses and durability, while process maturity determines reproducibility, cost, and scalability [97,98,99,100].

The discussion below links these system-level barriers to the material and manufacturing factors that produce them. Printable conductive materials and surface quality affect power density and internal resistance; durability and interface stability govern long-term operation; and material-process compatibility, dimensional control, and process maturity determine reproducibility, cost, scalability, and standardization. Evaluation under variable real-world conditions further reinforces the need to move beyond short-term proof-of-concept demonstrations.

### 7.1. Printable Conductive Materials

One of the principal limitations of current AM technologies is the relatively limited availability of materials capable of simultaneously satisfying the electrical, mechanical, chemical, and manufacturing requirements of electrochemical devices [97,98,99,100]. This challenge is particularly relevant because the advantages offered by geometric design freedom can only be fully exploited when suitable materials are available to provide the required functionality.

Electrochemical components such as bipolar plates, current collectors, porous electrodes, and conductive supports require a demanding combination of properties, including high electrical conductivity, corrosion resistance, mechanical integrity, and long-term chemical stability under operating conditions. Although a growing range of printable polymers, metals, ceramics, and composite materials is commercially available, relatively few materials combine all these properties while remaining compatible with reliable and scalable AM processes.

Conductive polymer composites represent a particularly promising approach because they combine low density, functional tunability, and manufacturing flexibility. However, achieving sufficiently high electrical conductivity often requires substantial concentrations of conductive fillers, such as graphite, carbon black, carbon nanotubes, or graphene. High filler loadings can adversely affect rheological behavior, printability, dimensional accuracy, interlayer adhesion, and mechanical properties, creating important trade-offs between processability and functional performance. Metallic AM can provide substantially higher electrical conductivity and mechanical strength, but typically involves higher material and equipment costs, more demanding processing conditions, and additional post-processing requirements.

In this context, Materials Science and Engineering plays a central role in maximizing the potential of AM as a design enabler for electrochemical devices. Progress requires not only the identification of materials compatible with existing AM processes, but also the development of new materials, composites, and processing strategies specifically tailored to the multifunctional requirements of electrochemical applications. Establishing clear relationships among composition, processing conditions, resulting microstructure, and functional properties will be essential for translating AM-enabled designs into reliable devices.

Therefore, future research should extend beyond the development of new printable materials toward systematic comparative studies of material–process–structure–property–performance relationships. Such studies should clarify how material selection and AM processing parameters determine microstructure and, in turn, how these characteristics affect electrical, mechanical, transport, interfacial, and electrochemical performance. This integrated materials-and-processing perspective will be critical for selecting appropriate material–process combinations and accelerating the transition of AM-enabled electrochemical architectures from laboratory prototypes to scalable and durable devices.

### 7.2. Resolution and Surface Finish

Although AM enables unprecedented geometric complexity, the achievable dimensional accuracy, resolution, and surface quality depend strongly on the selected AM process and technology, and not all approaches can provide tolerances or surface finishes comparable to those achievable with certain conventional manufacturing techniques [97,98,99,100]. These limitations can, however, be mitigated through a wide range of post-processing treatments, including mechanical operations such as grinding and polishing, surface coatings, and thermal treatments. Such procedures can be specifically tailored to relieve residual stresses, reduce part distortion and surface roughness, decrease surface-connected porosity, and improve mechanical and corrosion properties. Nevertheless, these additional processing steps increase manufacturing time, complexity, and cost.

Electrochemical performance can be particularly sensitive to geometric tolerances and surface characteristics. Small deviations in channel dimensions, pore morphology, or surface roughness can significantly affect reactant transport, pressure losses, electrical contact, and water management. Similarly, rough or irregular printed surfaces may increase interfacial contact resistance or modify local flow conditions, introducing additional variability into device performance. Reliable inspection and qualification of AM components are therefore particularly important, especially for complex geometries containing internal features that cannot be evaluated through conventional surface measurements. Non-destructive testing (NDT) techniques such as X-ray computed tomography (CT) are especially valuable in this context, as they enable three-dimensional inspection of both external geometry and internal features, including channels, pores, defects, and other geometrical characteristics of AM-fabricated components.

Continued advances in printing resolution, dimensional control, surface quality, in situ process monitoring, NDT, and component qualification methodologies will therefore be essential for the reliable fabrication of electrochemical components. In particular, establishing robust quality-control procedures capable of linking process conditions with dimensional accuracy, internal integrity, and functional performance will be critical for ensuring repeatability and reproducibility across AM cycles and achieving the precision required for high-performance electrochemical applications.

### 7.3. Long-Term Durability

Demonstrating the long-term reliability of additively manufactured electrochemical components remains another important challenge. Most published studies have focused on proof-of-concept demonstrations or short-term performance evaluation, whereas practical devices must operate reliably over thousands of hours under chemically and mechanically demanding conditions [97,98,99,100,121,122,123].

Repeated thermal cycling, humidity fluctuations, mechanical stresses, corrosion, and electrochemical degradation may affect printed materials differently from conventionally manufactured components because AM often produces anisotropic microstructures and process-dependent defects.

The long-term stability of interfaces between printed layers, conductive pathways, and multifunctional integrated components therefore requires systematic investigation. Standardized durability protocols specifically developed for additively manufactured electrochemical devices would facilitate meaningful comparisons between different manufacturing technologies and accelerate industrial adoption.

### 7.4. Manufacturing Scalability

Although AM excels during research, prototyping, and low-volume production, its economic competitiveness for large-scale manufacturing remains an open question [97,98,99,100]. Many current AM processes involve relatively slow build rates compared with established industrial techniques such as injection molding, metal stamping, or roll-to-roll manufacturing. Consequently, the advantages of geometric complexity must be balanced against production throughput, material utilization, equipment costs, and overall manufacturing economics.

Rather than replacing conventional manufacturing entirely, AM may initially be adopted in hybrid manufacturing strategies. Complex multifunctional components could be fabricated additively, while simpler high-volume parts continue to be produced using established industrial processes. Such hybrid approaches may offer practical pathways toward industrial implementation while preserving the unique design advantages provided by AM.

As printing technologies continue to mature, improvements in productivity, automation, and process reliability are expected to increase their competitiveness for broader electrochemical manufacturing applications.

### 7.5. Multi-Material Printing

One of the most transformative opportunities offered by AM—and simultaneously one of its greatest technical challenges—is the development of reliable multi-material manufacturing strategies [97,98,99,100]. Combining different materials within a single additively manufactured component makes it possible to integrate complementary properties that are difficult to achieve with a single material. For example, bimetallic architectures can combine corrosion resistance, strength, and thermal performance, whereas metal–polymer combinations can provide lightweight structures with efficient heat dissipation and tailored mechanical behavior.

This capability is particularly attractive for electrochemical devices, which inherently combine materials with very different functions, including electrically conductive elements, ion-conducting membranes, porous electrodes, catalytic layers, structural supports, sealing components, and thermal-management features. Conventional manufacturing typically produces these elements separately and subsequently joins them through multiple fabrication and assembly operations. In contrast, several AM technologies are being developed to integrate dissimilar materials within a common manufacturing workflow. Directed energy deposition (DED), for example, can fabricate bimetallic structures by combining different metallic alloys and creating graded transitions in composition and properties [124]. Metal–polymer architectures can likewise be produced through approaches such as overprinting and advanced joining techniques [125], enabling increasingly integrated multifunctional components.

Future multi-material AM systems could enable several of these functional materials to be deposited or integrated within a single manufacturing sequence, reducing component count and assembly requirements while improving structural cohesion and functional integration. Such approaches could ultimately support electrochemical devices in which structural, electrical, thermal, and electrochemical functions are co-designed and manufactured rather than assembled from individually fabricated parts.

Achieving this vision, however, requires precise control over material compatibility, interfacial bonding, deposition sequence, thermal history, and dimensional accuracy. Differences in viscosity, curing behavior, melting temperature, thermal expansion, and chemical and electrochemical stability can complicate the processing of multiple materials and may introduce residual stresses, weak interfaces, or property gradients that affect long-term device performance.

Overcoming these challenges will require close integration of materials science, process development, and computational design to establish robust relationships among composition, manufacturing conditions, interfacial microstructure, and functional behavior. Success in this area would represent a major step toward highly integrated electrochemical devices in which structural, electrical, thermal, and electrochemical functions are manufactured within coordinated workflows.

### 7.6. Life-Cycle and Economic Competitiveness

Practical adoption also requires AM-enabled architectures to be competitive over both their economic and environmental life cycles. At low production volumes, AM can avoid dedicated tooling and reduce the cost of design iteration, while part consolidation may reduce assembly operations, inventories, and interface-related failures. At higher volumes, however, conventional stamping, molding, or machining can retain strong advantages in throughput and unit cost. The relevant comparison should therefore include machine capital and utilization, feedstock price, build rate, yield, powder recovery, support removal, heat treatment, surface finishing, inspection, and rejected-part rates rather than comparing raw material use alone.

The same system boundary is required for life-cycle assessment. Material savings and part consolidation can be offset by electricity demand, inert-gas use, support material, powder losses, and post-processing, as discussed in Section 1 [38]. For electrochemical devices, the use phase must also be considered: an AM component with a higher manufacturing burden may still provide a net benefit if improved transport or lower resistance produces sufficient efficiency, durability, mass, or balance-of-plant savings during operation. Future comparisons should therefore report cost per functional component together with energy and material inputs, production volume, lifetime, and performance delivered over that lifetime. Such functional-unit-based comparisons are more informative than treating AM as intrinsically more or less sustainable than conventional manufacturing.

Taken together, the challenges in Figure 9 show that progress cannot be judged solely by the ability to print increasingly complex geometries. AM-enabled components must also deliver competitive power density and low internal resistance, retain functionality during prolonged operation, overcome material and process limitations, and be manufactured reproducibly at acceptable scale and cost. For the specific open columnar cathode plate considered here, the most immediate barrier to translation is scalable and reproducible manufacturing at competitive cost. The laboratory-scale electrochemical benefit has been demonstrated, but practical deployment will require the L-PBF process to maintain dimensional accuracy, surface quality, electrical contact, and part-to-part consistency while reducing build time and post-processing requirements. Long-term durability remains essential, but manufacturing reproducibility and cost are likely to determine whether the architecture can progress from a successful laboratory component to a practical production route. Standardized characterization and qualification under realistic environmental and operating conditions will therefore be essential for comparing technologies and building confidence beyond laboratory demonstrations.

These requirements mark the transition from geometric feasibility to practical implementation. They also provide a direct bridge to Section 8: the future research directions summarized there can be understood as responses to the barriers in Figure 9, with advances in materials, computational design, system integration, scalable manufacturing, and digital workflows addressing different parts of the same translation challenge.

## 8. Future Research Directions

Future work should move from demonstrating printable complexity toward testing explicit, falsifiable design hypotheses. The priorities below are therefore framed around measurable research questions: which geometric variables control performance, how can they be optimized under manufacturing constraints, how robust are the gains across operating conditions and orientations, and at what production scale and lifetime do they justify the added manufacturing burden? For portable air-breathing devices, progress should be evaluated simultaneously against electrochemical performance, inactive mass and volume, durability, reproducibility, and cost.

Figure 10 organizes these opportunities into six complementary directions: advanced materials; computational design and AI; system integration; digital design and AM workflows; scalability and manufacturing; and extension beyond conventional PEMFC architectures, including flexible devices. The subsections below develop specific elements of this framework. AI, topology optimization, and bio-inspired design expand the computational design space; embedded sensing and multifunctional printing strengthen system integration; and digital twins connect physical devices with models and feedback. Advances in materials and scalable manufacturing underpin these directions. Importantly, they should not be regarded as independent pathways. Their convergence could establish closed-loop design–manufacture–test workflows in which experimental feedback continuously informs computational optimization and fabrication, accelerating the transition from proof-of-concept geometries to robust, application-specific electrochemical devices.

### 8.1. AI-Driven Electrochemical Design

AI is expected to play an increasingly important role in electrochemical-device development by accelerating the exploration of design spaces that are difficult to investigate experimentally. This transition has already begun in PEMFC flow-field design. Zhou et al. combined CFD data with a neural-network surrogate to screen tens of thousands of candidate flow fields and experimentally validated an optimized geometry with a 10.37% increase in peak power density relative to a serpentine reference [103]. Such examples show that AI-assisted design can move from performance prediction to the selection of experimentally testable electrochemical architectures. A near-term priority is to couple such surrogate models to experimentally measured uncertainty and manufacturability constraints so that selected designs are not only numerically optimal but also reproducible after printing.

Machine-learning models trained on experimental measurements and numerical simulations can rapidly identify relationships between geometric parameters and electrochemical performance. Such models may predict the influence of architectural modifications on reactant transport, thermal behavior, electrical resistance, or water management, allowing promising designs to be identified before fabrication.

Beyond predictive modeling, AI-assisted optimization can automate portions of the design process by proposing geometries that satisfy multiple performance objectives simultaneously. As these methods mature, the interaction between computational optimization and AM is likely to reduce development time while expanding the range of feasible electrochemical architectures.

### 8.2. Topology Optimization and Generative Design

One of the greatest opportunities created by AM is the application of topology optimization and generative design to electrochemical components [94,97,98,99,100,101,126]. Rather than beginning from a predefined channel pattern, topology optimization can allow material and fluid domains to evolve according to objectives such as reactant transport, pressure loss, current-density uniformity, thermal management, or mechanical integrity. For PEMFC cathode gas channels, Xia et al. demonstrated this approach using a transport-aware topology-optimization framework that generated non-conventional channel architectures with improved predicted performance [102]. AM is particularly relevant to this workflow because it can provide a practical route for fabricating geometries that are difficult to realize by machining or forming. Future studies should therefore perform multi-objective optimization in which electrochemical gain is penalized explicitly by pressure loss, inactive mass, minimum printable feature size, surface-finish requirements, and post-processing accessibility, followed by experimental comparison against a common baseline.

Traditional engineering design generally begins with predefined geometries that are modified iteratively according to performance requirements. In contrast, topology optimization determines the optimal material distribution within a prescribed design domain while satisfying constraints related to mechanical strength, fluid transport, thermal management, electrical conductivity, or manufacturing feasibility.

Generative design extends this concept by automatically producing numerous candidate architectures that can be evaluated using multiphysics simulations and optimization algorithms. When coupled with AM, these computational approaches enable the manufacturing of highly complex geometries that would be impractical to fabricate using conventional methods.

Future electrochemical devices may therefore evolve from manually designed structures toward algorithm-generated architectures specifically tailored to their operating conditions.

### 8.3. Bio-Inspired Architectures and Flexible Devices

Natural transport systems have evolved over millions of years to distribute fluids, nutrients, heat, and structural loads with remarkable efficiency. Biological structures such as vascular networks, leaf venation, trabecular bone, coral formations, and branching trees provide valuable inspiration for electrochemical architectures in which reactant distribution and multifunctionality are critical [97,100].

AM offers the geometric flexibility required to reproduce many of these complex hierarchical structures. Bio-inspired airflow pathways, porous electrodes, lattice frameworks, and branching manifolds may enhance oxygen transport, facilitate passive water removal, improve heat dissipation, and reduce material consumption simultaneously.

Combining biological inspiration with computational optimization may enable electrochemical devices that achieve higher performance through improved transport efficiency rather than solely through advances in electrochemical materials.

Flexible devices can accommodate bending and conform to changing geometries with minimal variation in essential functional parameters, facilitating integration and operation in portable applications and biological environments such as skin, the human body, and clothing. AM methods using flexible polymers for structural bodies and plates, combined with low-resistance interfaces and locally reinforced regions, could enable electrochemical devices that combine mechanical flexibility with the robustness required for reliable operation.

### 8.4. Embedded Sensors and Smart Electrochemical Devices

Future additively manufactured electrochemical systems are likely to incorporate sensing capabilities directly within structural components. Advances in printable conductive materials and embedded electronics may enable the integration of temperature sensors, strain gauges, humidity sensors, pressure sensors, or electrical monitoring elements during the manufacturing process [97,98,99,100].

Embedded sensing would allow continuous monitoring of operating conditions, enabling early detection of performance degradation, water accumulation, thermal hotspots, or mechanical damage. Such information could support adaptive operating strategies, predictive maintenance, and improved long-term reliability.

For portable systems operating under variable environmental conditions, integrated sensing may become an important component of intelligent energy management and autonomous system control.

### 8.5. Multifunctional Printed Devices

A particularly promising direction for future research is the development of multifunctional printed components that simultaneously perform structural, electrical, thermal, and electrochemical functions [97,98,99,100].

Rather than assembling numerous discrete components, future electrochemical devices may incorporate current collectors, flow distributors, structural reinforcements, seals, and thermal-management features within unified printed architectures. Multi-material AM will be essential for developing such integrated systems, enabling different functional materials to be deposited selectively according to local design requirements and thereby maximizing overall device performance.

This approach has the potential to reduce component count, simplify assembly, decrease manufacturing variability, and improve overall system reliability while maintaining compact and lightweight designs.

### 8.6. Digital Twins and Closed-Loop Development

The integration of AM with digital twins represents another promising research direction. A digital twin is a virtual representation of a physical system that continuously incorporates experimental data to improve predictive accuracy throughout the product lifecycle [97,98,99,100,127].

For electrochemical devices, digital twins could combine multiphysics simulations, manufacturing information, embedded sensor data, and operational measurements to predict performance, diagnose degradation mechanisms, and optimize operating conditions in real time.

Coupled with AM, this capability would enable rapid redesign and iterative improvement based on observed performance, creating a closed-loop development framework in which computational models, manufacturing processes, and experimental validation continuously inform one another. A practical milestone would be a closed-loop experiment in which in situ sensor data update a reduced-order model and trigger a geometry revision whose performance gain is then verified in the next printed generation.

### 8.7. Scale-Aware and Durability-Aware Design

Future optimization should include production and lifetime constraints from the outset. For the open columnar cathode plate, this means quantifying how dimensional tolerances, roughness, electrical contact resistance, build orientation, batch-to-batch variability, and post-processing affect both initial performance and degradation. Scale-up studies should report build time, yield, material utilization, inspection burden, and cost per plate as a function of batch size. The objective is not simply to reproduce laboratory performance at larger scale, but to identify the point at which geometric benefit remains significant after manufacturing variability, durability, and cost are included.

### 8.8. Toward Fully Printed Electrochemical Systems

The long-term vision of AM extends beyond the fabrication of individual components. Continued advances in printable functional materials, multi-material deposition, and process integration may eventually enable the production of fully printed electrochemical systems [97,98,99,100].

Although this objective remains technologically ambitious, future manufacturing platforms may fabricate structural elements, conductive pathways, porous electrodes, flow fields, seals, sensors, and other functional components within a single automated workflow. Such an approach would fundamentally redefine electrochemical manufacturing by minimizing assembly operations and maximizing architectural integration.

This multidisciplinary vision will require close collaboration among electrochemists, materials scientists, mechanical engineers, manufacturing specialists, computational designers, and AI researchers. Progress is therefore expected to depend not only on advances within individual disciplines but also on increasingly interdisciplinary approaches to electrochemical device development.

Collectively, the directions in Figure 10 form a connected development pathway rather than a set of independent topics. Advanced materials determine which functionalities can be realized with available AM technologies; computational design and AI identify architectures that exploit those materials; system integration combines structural, electrical, sensing, and transport functions; and digital workflows connect simulation, fabrication, testing, and redesign. Scalability will determine whether these advances can move beyond prototypes, while application to technologies beyond PEMFCs will test the generality of the design philosophy. This integrated view leads to the central conclusion of this perspective: AM is most powerful when treated as a platform for digitally driven electrochemical design rather than as an isolated manufacturing step.

## 9. Conclusions

Portable air-breathing electrochemical devices impose tightly coupled requirements on reactant transport, thermal regulation, mechanical robustness, and system integration. Throughout this perspective, geometry has emerged as the common variable linking these requirements, particularly in passive systems that must minimize mass, volume, and auxiliary hardware. Conventional manufacturing has often restricted this variable, causing practical architectures to reflect a compromise between electrochemical function and manufacturability.

AM changes that relationship by expanding the accessible design space. Its geometric freedom, capacity for functional integration, rapid prototyping, and compatibility with digital workflows allow architecture to be optimized alongside advanced materials, operating conditions, and electrochemical performance. The significance of AM is therefore not simply that it can fabricate complex parts, but that it permits different questions to be asked during design.

The open columnar cathode plate case study provides the central experimental validation of this design philosophy. By using AM to redistribute structural material according to local mechanical and transport functions, the architecture improves passive reactant access and water management while reducing inactive mass. Experimentally, the open configuration increases peak power density by approximately 56% relative to the reference columnar configuration and raises the reported mass-specific power-density metric from about 0.5 to 1.5 mW cm^−2^ g^−1^. The case study therefore demonstrates that AM can serve as a vehicle for both designing and manufacturing an architecture with improved electrochemical performance, rather than simply reproducing a conventionally conceived component. More broadly, it validates the principle that manufacturing-enabled geometry can improve the operating environment of an electrochemical device without changing its electrochemically active materials.

That principle can be extended beyond PEMFCs to metal–air batteries, microbial fuel cells, DMFCs, water electrolyzers, CO_2_ electrochemical reactors, and electrochemical sensors. Although the relevant transport processes and design objectives differ among these technologies, each can benefit from architectures that deliberately coordinate reactant pathways, product removal, electrical conduction, thermal management, and structural support. Related work on porous transport-layer/catalyst-layer interfaces in polymer-electrolyte electrolysis likewise highlights the importance of architecture-controlled transport and interfacial behavior.

Translation to practical devices nevertheless requires progress in printable conductive materials, dimensional accuracy and surface quality, long-term durability, scalable production, standardization, and multi-material fabrication. These are not separate from the design opportunity; they define the constraints within which AM-enabled architectures must ultimately operate and therefore need to be incorporated into future optimization workflows.

Looking forward, the convergence of AM with computational modeling, topology optimization, machine learning, sensing, and digital twins offers a route to increasingly closed-loop development. Geometry can be generated and evaluated computationally, fabricated rapidly, tested under realistic conditions, and refined using experimental data. Such workflows could shorten development cycles while allowing electrochemical, mechanical, thermal, and manufacturing objectives to be considered simultaneously.

The next generation of portable electrochemical devices will therefore depend not only on improved catalysts, membranes, and functional materials, but also on architectures that use those materials more effectively. The open columnar PEMFC cathode demonstrates the central proposition of this perspective: when manufacturing freedom is incorporated from the outset as a design variable, AM can enable architectures that deliver measurable electrochemical and system-level benefits. The broader opportunity is to transfer this function-driven approach to other electrochemical devices while respecting their technology-specific mechanisms, metrics, and manufacturing constraints. The central contribution of AM is thus not complexity for its own sake, but its capacity to connect functional design with manufacturable geometry and thereby support lighter, more compact, more efficient, flexible, and highly integrated electrochemical systems.

## Figures and Tables

**Figure 1:**
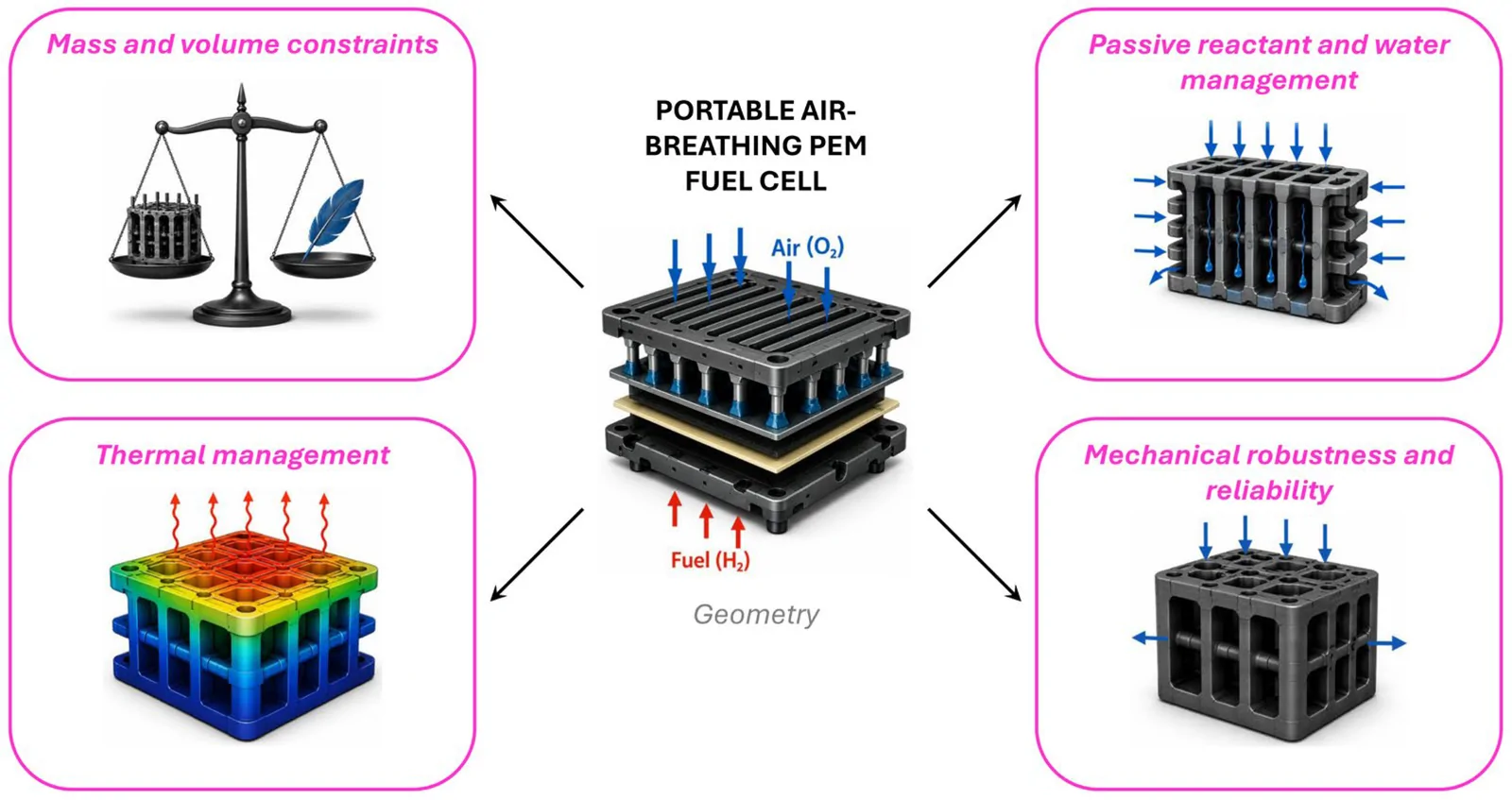
Key design requirements for portable air-breathing electrochemical devices. Schematic representation of the coupled constraints governing device architecture, including mass and volume minimization, passive reactant and water management, thermal regulation, and mechanical robustness. Illustrative images were generated using AI.

**Figure 2:**
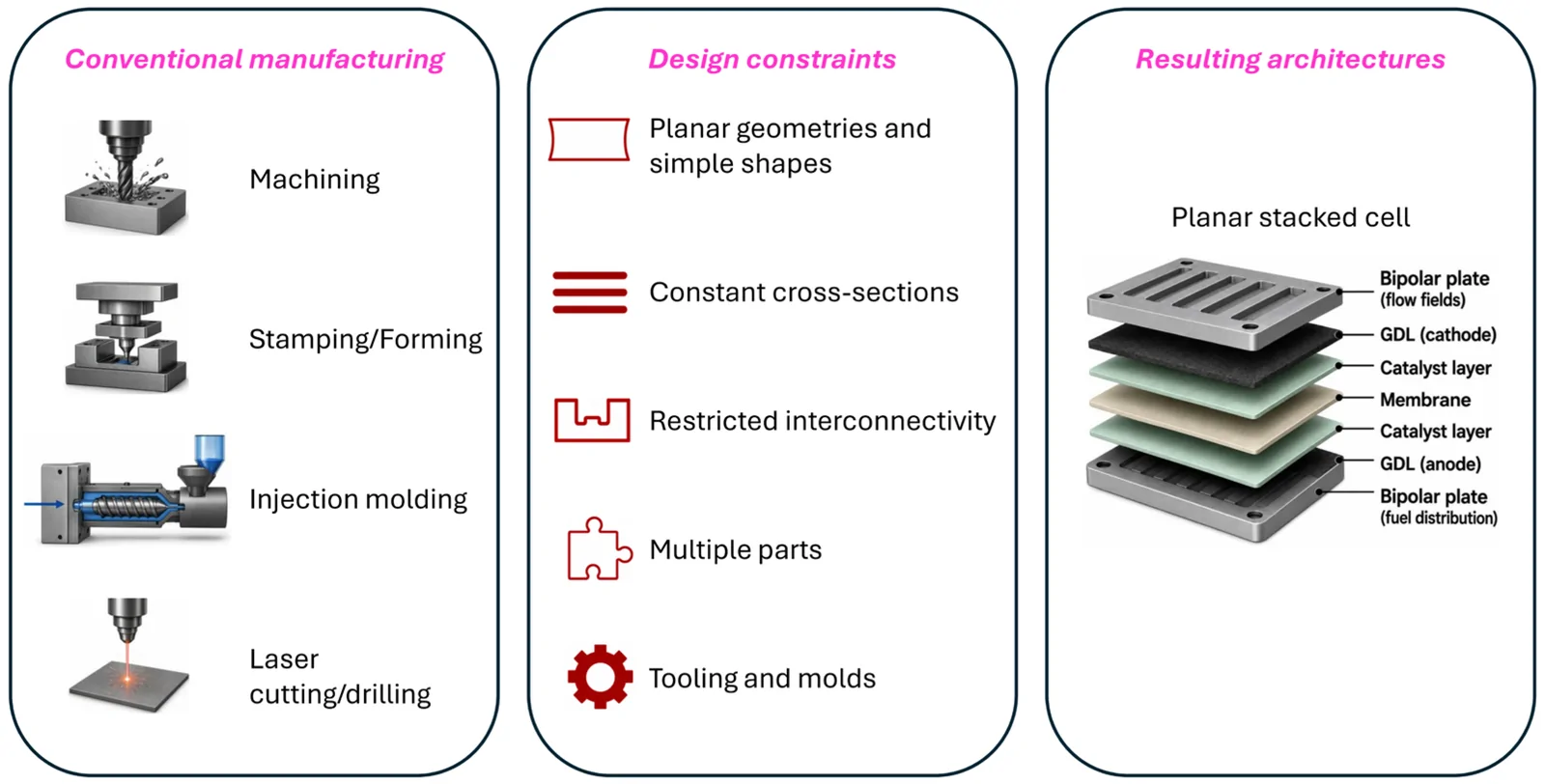
Manufacturing constraints shaping conventional electrochemical device architectures. Schematic representation of established fabrication methods, including machining, metal forming, injection molding, and laser cutting, favor relatively simple and predominantly planar geometries; by limiting three-dimensional transport pathways, functional integration, and architectural optimization, which motivates the transition toward manufacturing strategies with greater geometric freedom. Illustrative images were generated using AI.

**Figure 3:**
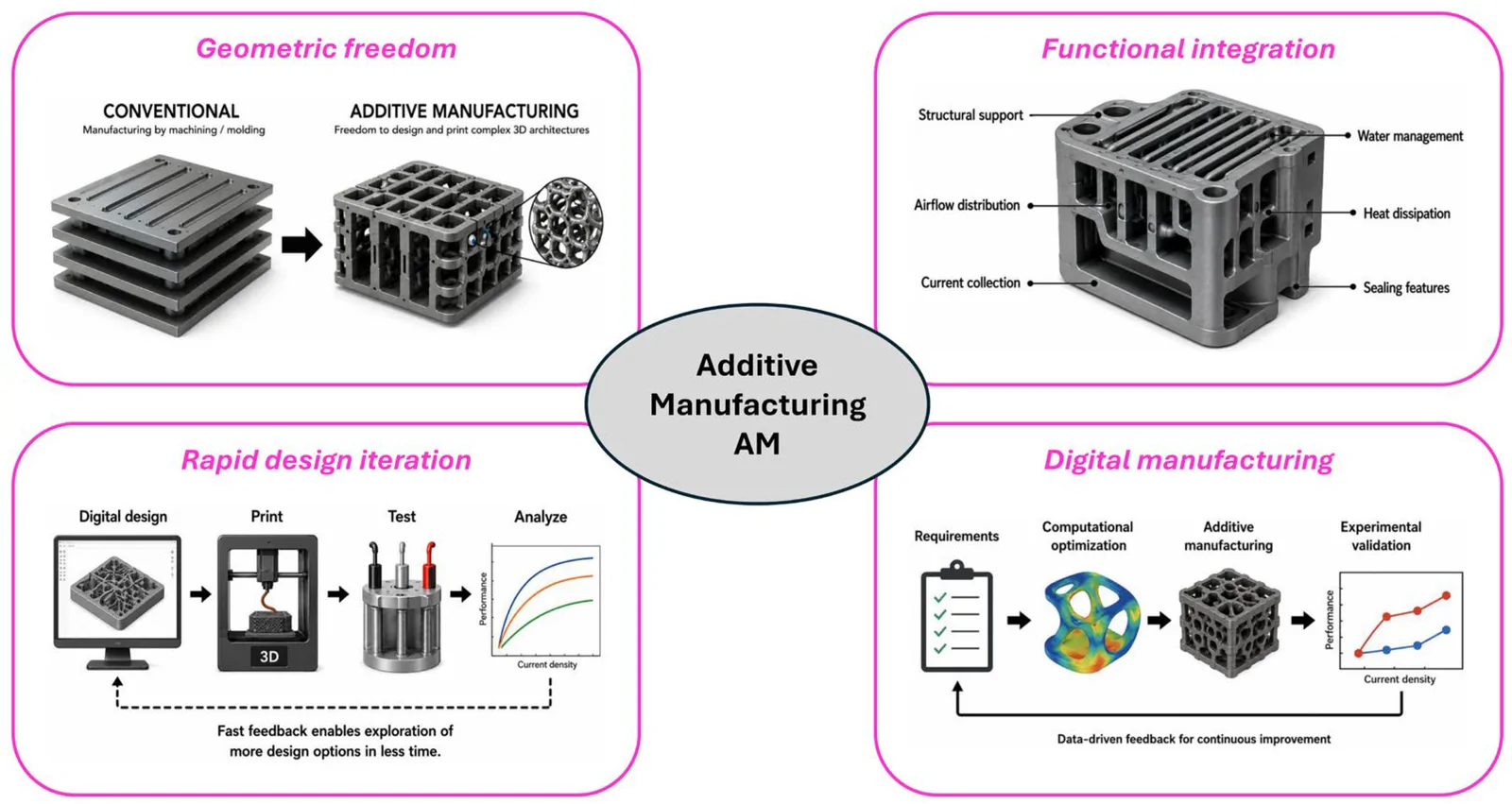
AM as a holistic design enabler for electrochemical devices. Schematic representation of the main capabilities offered by AM, including geometric freedom for complex three-dimensional architectures, functional integration of structural, electrical, fluidic, thermal, and sealing features, rapid design–print–test iteration, and integration with computational optimization and digital manufacturing workflows. Together, these capabilities expand the accessible design space and enable electrochemical architectures to be optimized according to their functional and transport requirements rather than conventional manufacturing constraints. Illustrative images were generated using AI.

**Figure 4:**
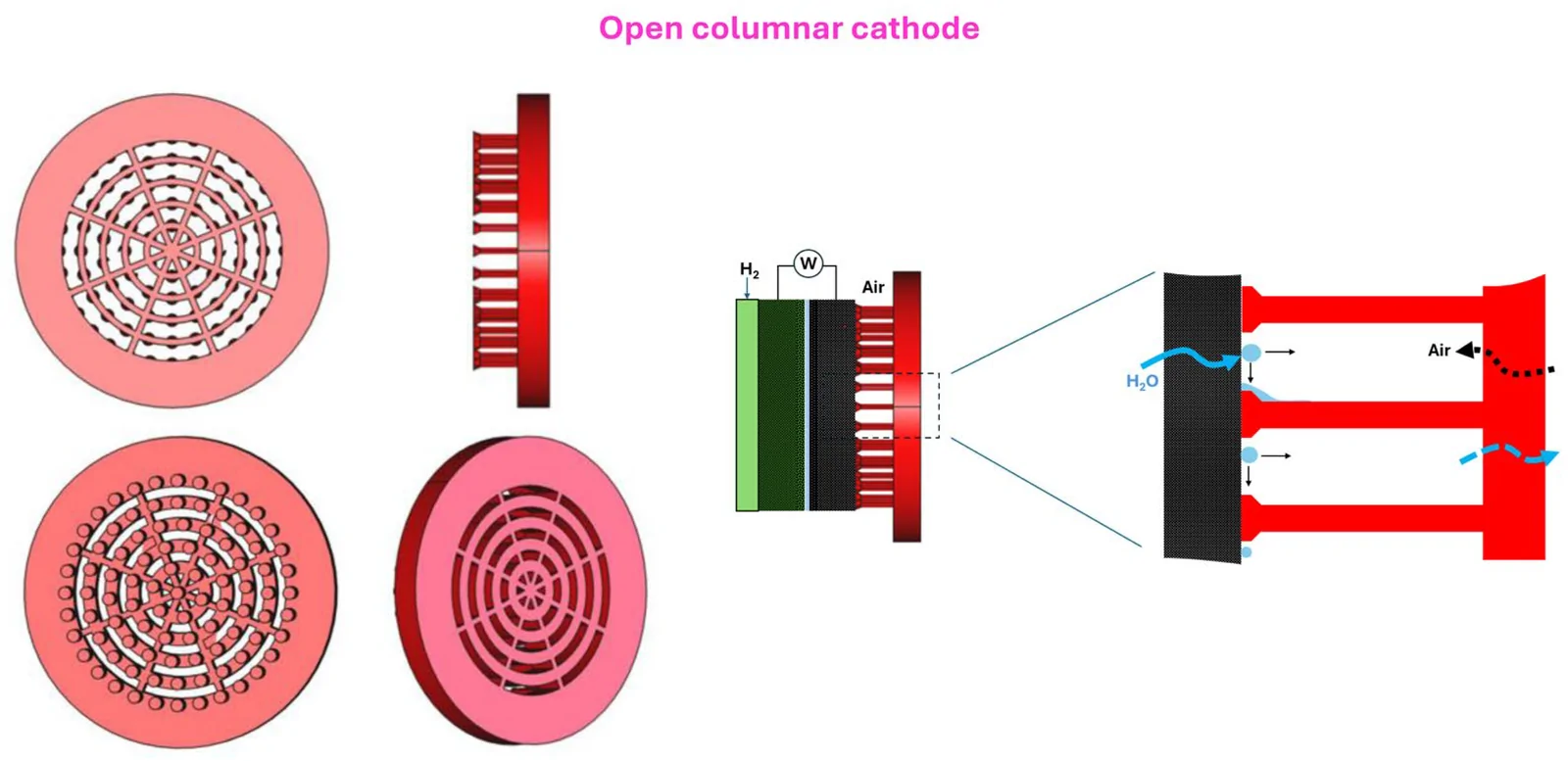
(**Left**) Open columnar cathode architecture for a passive air-breathing PEMFC. The slotted cathode plate combines slender columns with enlarged distal contact regions to maintain mechanical compression and electrical contact while increasing the open volume available for passive air access and water removal. (**Right**) Scheme (not to scale) of the plate assembled at the air-breathing cathode side of a hydrogen PEMFC. The architecture illustrates the integration of structural, electrical, and mass-transport functions through geometric design.

**Figure 5:**
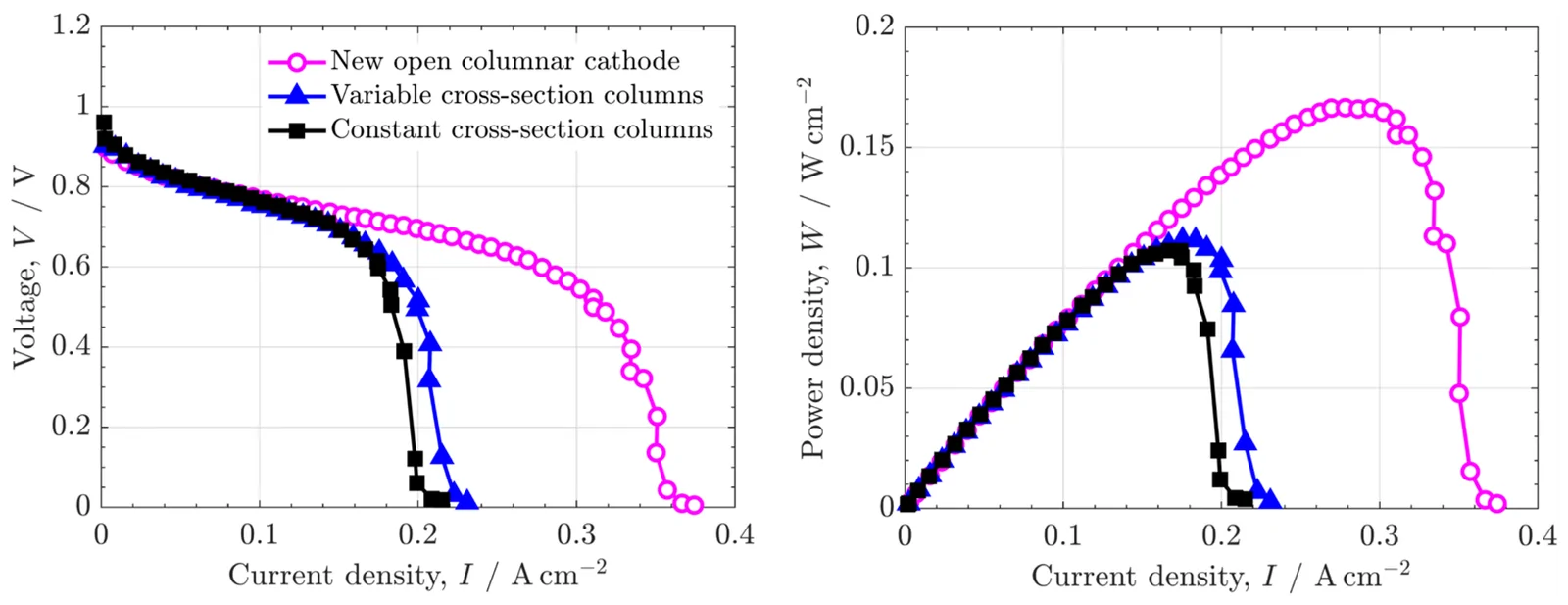
Effect of cathode architecture on the performance of passive air-breathing PEMFCs. Polarization (left) and power-density (right) curves compare three columnar cathode configurations: base plate with constant-section columns, modified columns with enlarged distal contact regions, and an open columnar architecture combining the modified columns with through-slots. Opening the supporting plate alleviates mass-transport limitations, extends the achievable current density, and increases the peak power density, demonstrating the performance benefits of geometric optimization.

**Figure 6:**
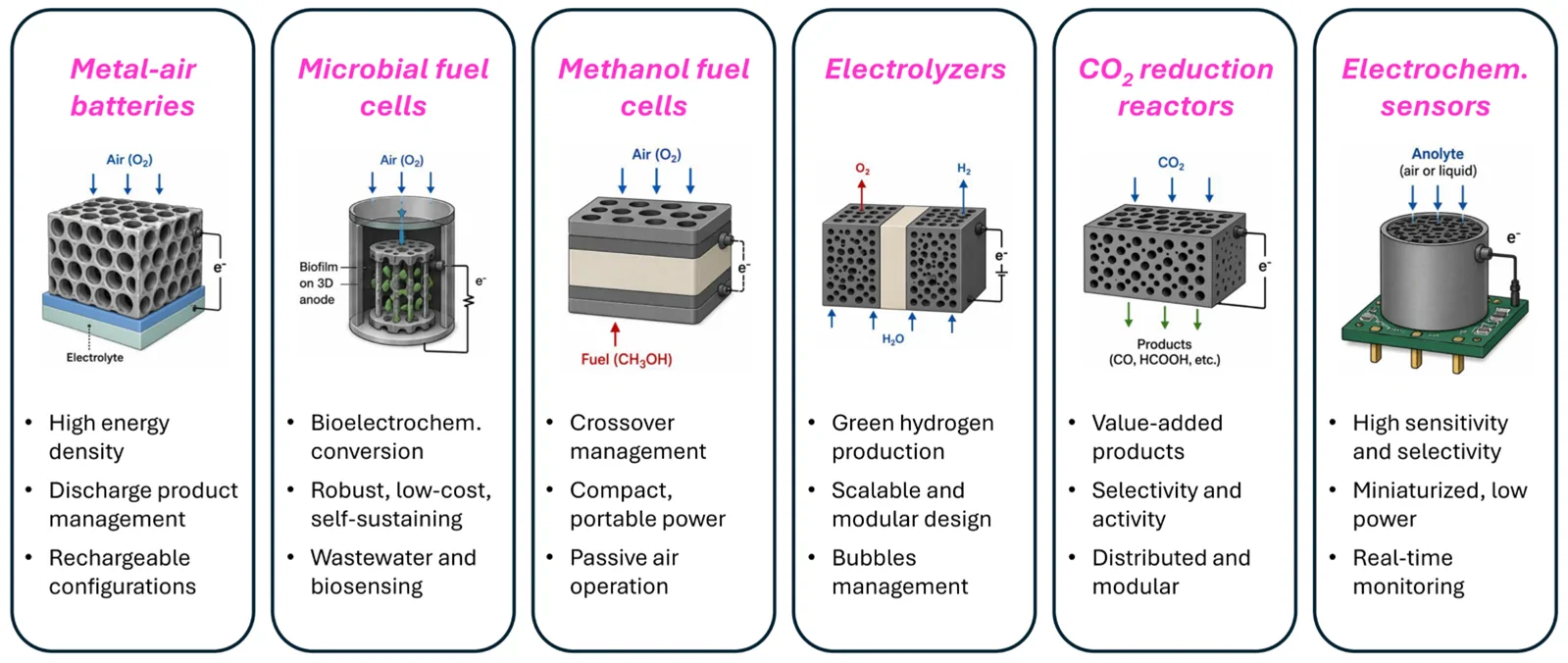
Generalization of AM-enabled design beyond PEMFCs. Schematic representation of the extension of geometry-driven design principles to diverse electrochemical technologies, including metal–air batteries, microbial fuel cells, direct methanol and other fuel cells, and electrochemical sensors. Despite differences in operating principles, these systems share opportunities to exploit AM for tailored three-dimensional architectures. Illustrative images were generated using AI.

**Figure 7:**
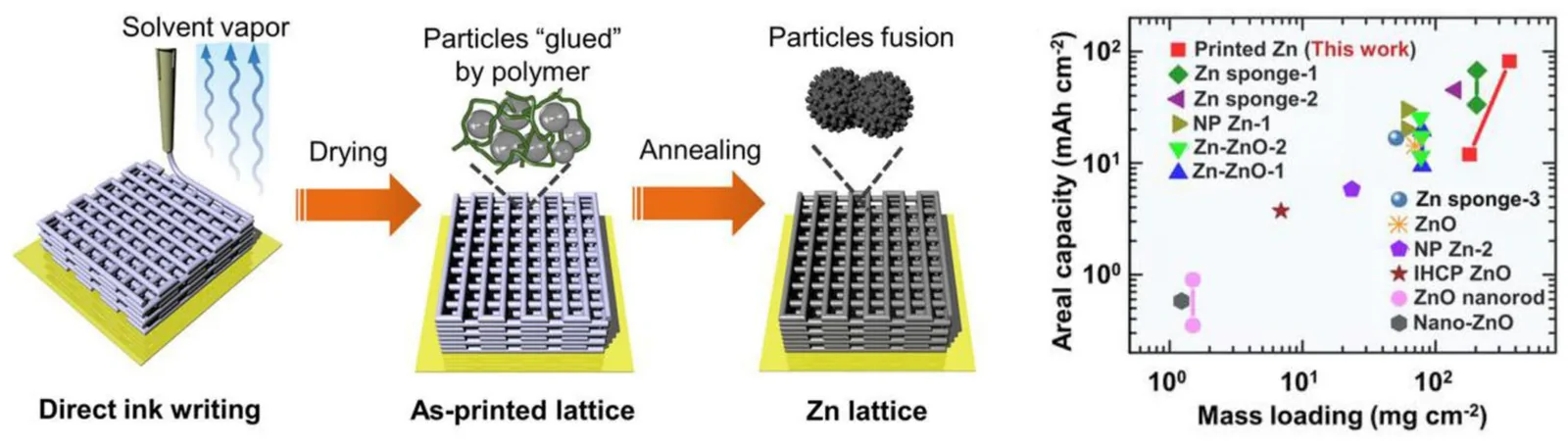
Additively manufactured three-dimensional Zn anodes for rechargeable alkaline batteries. (**Left**) direct-ink-writing and post-processing route used to produce a freestanding metallic Zn lattice. (**Right**) areal capacity as a function of Zn mass loading for the printed Zn architecture compared with previously reported Zn electrodes. The printed Zn lattice delivered an average areal capacity of 11.89 mAh cm^−2^ during more than 650 cycles at 25 mA cm^−2^, while a thicker printed architecture reached 85.45 mAh cm^−2^. Adapted from Zhu et al. [113] under the Creative Commons Attribution (CC BY) license.

**Figure 8:**
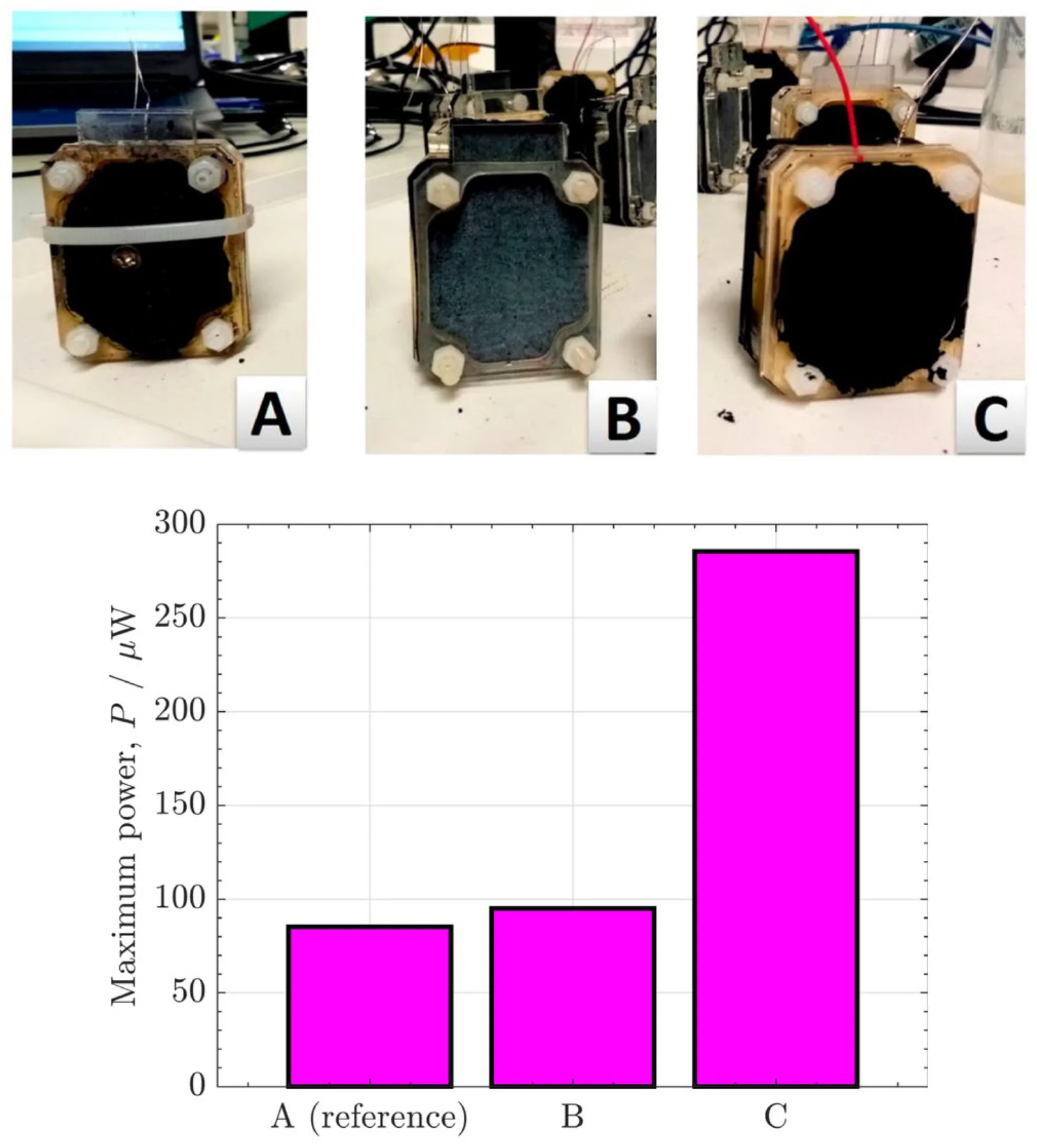
Quantitative performance benchmark for a microbial fuel-cell cathode. The AM-compatible PTFE-free activated-carbon cathode (**C**) produced 286 μW maximum power, compared with 98 μW for PTFE-AC (**B**) and 85 μW for the activated-carbon block reference (**A**). Data reproduced from Theodosiou et al. [117] under the Creative Commons Attribution (CC BY) license.

**Figure 9:**
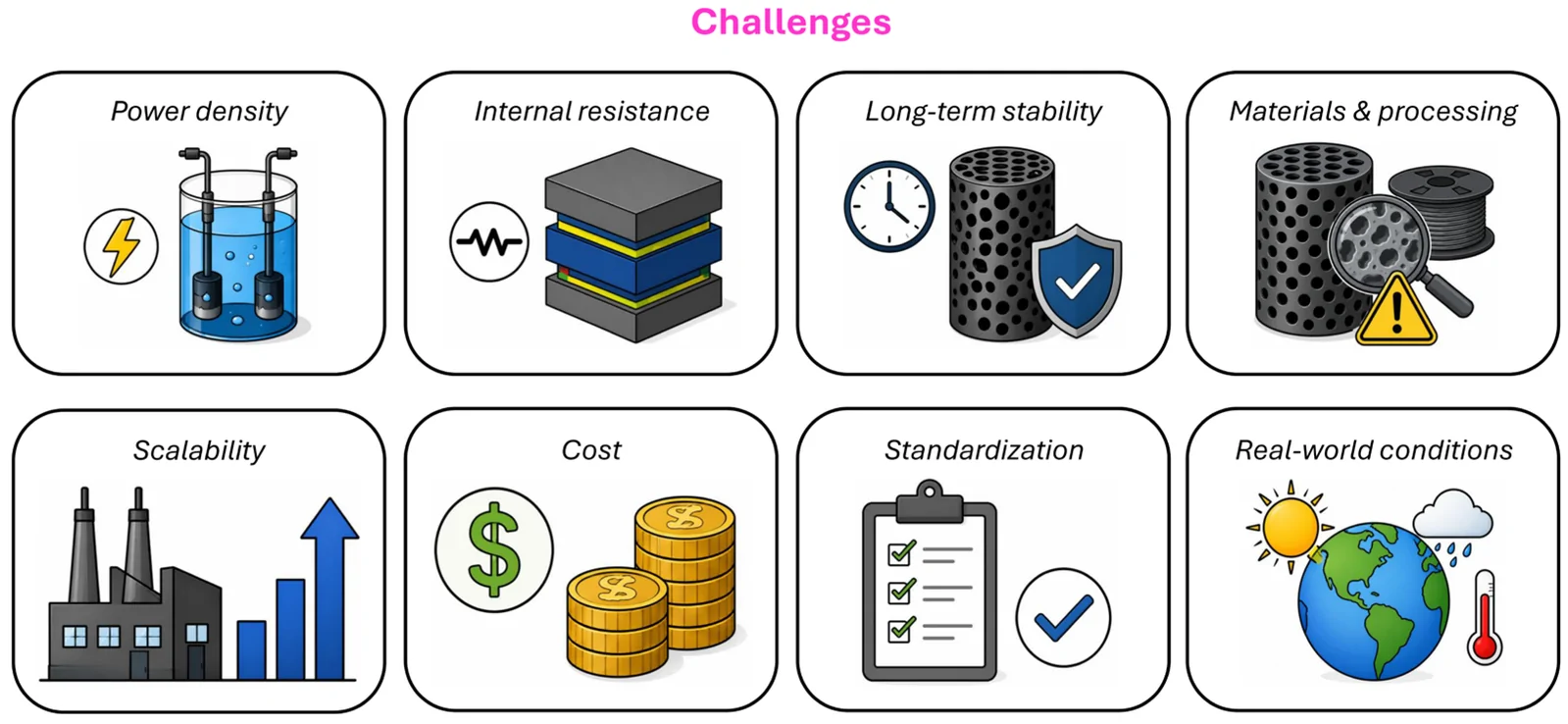
Remaining challenges for AM of electrochemical devices. The principal barriers span device performance and technology translation, including power density, internal resistance, long-term stability, material and process limitations, scalability, cost, standardization, and operation under real-world conditions. These challenges are coupled to printable-material properties, manufacturing precision, durability, process reproducibility, and the maturity of scalable fabrication routes. Illustrative images were generated using AI.

**Figure 10:**
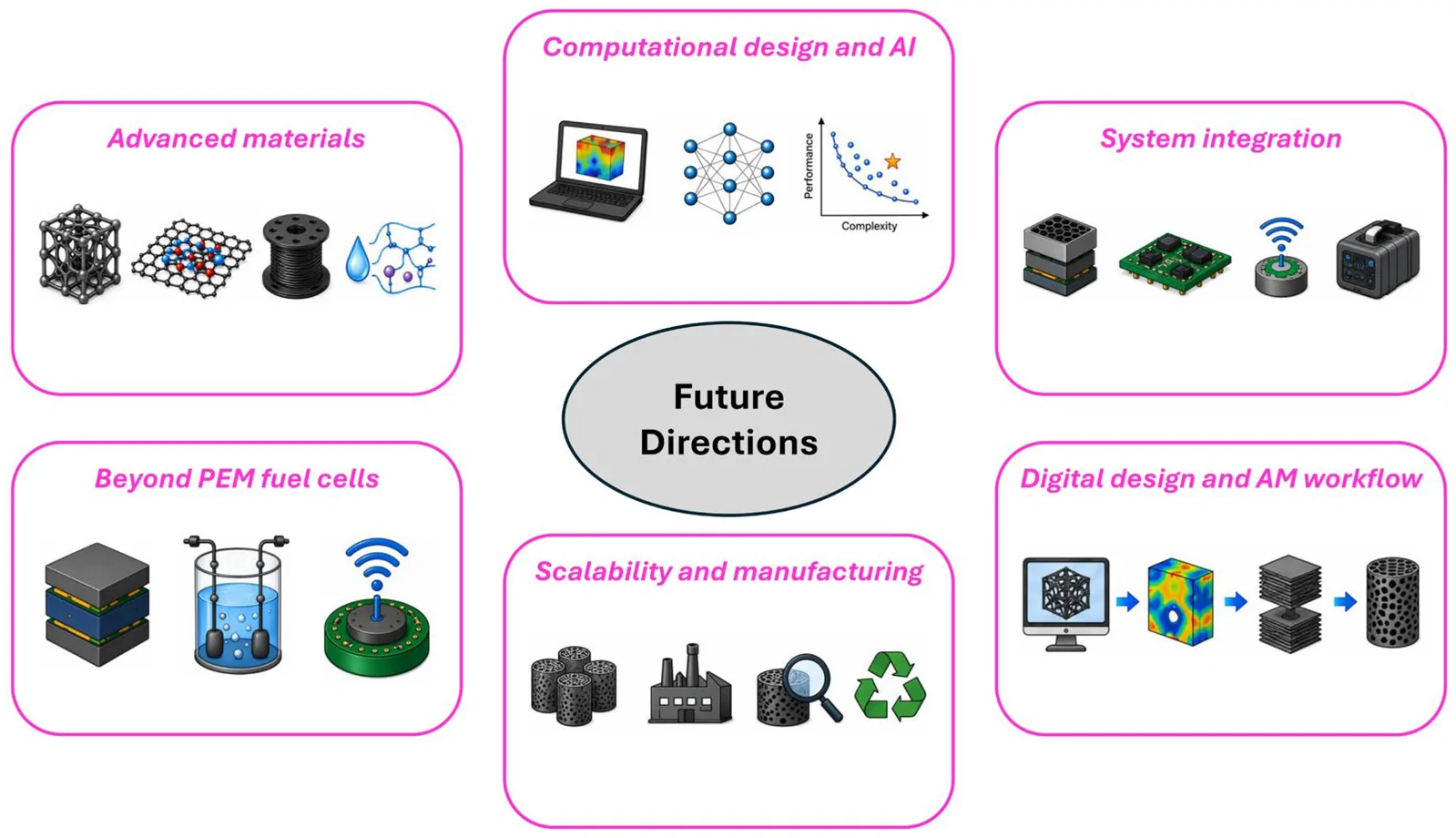
Future directions for AM of electrochemical devices. Six complementary research directions are highlighted: advanced materials, computational design and AI, system integration, digital design and AM workflows, scalability and manufacturing, and extension beyond PEMFCs. Their convergence can connect material development, architecture optimization, fabrication, sensing, validation, and redesign, accelerating the translation of AM-enabled concepts into integrated and application-specific electrochemical systems. Illustrative images were generated using AI.

## Data Availability

No new data were created or analyzed in this study. Data sharing is not applicable to this article.

## Abbreviations

The following abbreviations are used in this manuscript:

AI	Artificial intelligence
AM	Additive manufacturing
CT	Computed tomography
DED	Directed energy deposition
DMFC	Direct methanol fuel cell
L-PBF	Laser powder bed fusion
MEA	Membrane electrode assembly
NDT	Non-destructive testing
PEMFC	Polymer electrolyte membrane fuel cell
